# Rapid mapping of global flood precursors and impacts using novel five-day GRACE solutions

**DOI:** 10.1038/s41598-024-64491-w

**Published:** 2024-06-15

**Authors:** Ashraf Rateb, Himanshu Save, Alexander Y. Sun, Bridget R. Scanlon

**Affiliations:** 1 Bureau of Economic Geology, Jackson School of Geosciences, The University of Texas at Austin, Austin, TX 78758 USA; 2 Center for Space Research, University of Texas at Austin, Austin, TX 78759 USA

**Keywords:** Hydrology, Natural hazards

## Abstract

Floods affect communities and ecosystems worldwide, emphasizing the importance of identifying their precursors and enhancing resilience to these events. Here, we calculated Antecedent Total Water Storage (ATWS) anomalies from the new 5-day (5D) Gravity Recovery and Climate Experiment (GRACE) and its Follow-On (GRACE-FO) satellite solutions to enhance the detection of pre-flood and active flood conditions and to map post-flood storage anomalies. The GRACE data were compared with ~ 3300 flood events reported by the Dartmouth Flood Observatory (2002–2021), revealing distinct ATWS precursor signals in 5D solutions, in contrast to the monthly solutions. Specifically, floods caused by saturation-excess runoff—triggered by persistent rainfall, monsoonal patterns, snowmelt, or rain-on-snow events—show detectable ATWS increases 15 to 50 days before and during floods, providing a valuable opportunity to improve flood monitoring. These 5D solutions also facilitate a more rapid mapping of post-flood storage changes to assess flood recovery from tropical cyclones and sub-monthly weather extremes. Our findings show the promising potential of 5D GRACE solutions, which are still in the development phase, for future integration into operational frameworks to enhance flood detection and recovery, facilitating the rapid analysis of storage changes relative to monthly solutions.

## Introduction

Floods represented 43% of all natural disasters between 1998 and 2017, affecting ~ 2 billion people and causing economic losses of ~ $656 billion^1^. Floods also exacerbate indirect effects such as waterborne diseases, ecosystem damage, and disruption of supply chains^2,3^. The rising flood risk driven by increased short- and long-term rainfall extremes due to climate warming^4–7^ highlights the critical need to understand flood precursors for effective mitigation and flood recovery for management.

Advances in data have enhanced our understanding of flood causation^8^. Initial studies linked floods to weather systems and atmospheric oscillations^9–11^, focusing on moisture dynamics^12^. Subsequent research has investigated flood origins using variables such as soil moisture and runoff^8^. Recent studies have expanded to include various types of floods—such as those resulting from prolonged rainfall, snowmelt, and flash floods—categorized by their underlying causes and seasonal patterns^13,14^. The Dartmouth Flood Observatory (DFO) classifies floods based on their causes, including heavy rain, snowmelt, rain-on-snow, and tropical cyclones, utilizing both remote sensing data and ground reports^15^.

Flood vulnerability assessment integrates modeling, in-situ data, and remote sensing. Hydrodynamic and hydrological models, which rely on inputs from weather and climate models, simulate water flow^16^ and forecast runoff^17^, respectively, to assess flood frequency and intensity. Despite their benefits, these models have significant uncertainties in model parameters and external forcing, which affect their accuracy in predicting peak flood events. Addressing these challenges necessitates reliable precipitation data, event-specific model calibration, and efficient management of computational resources^18^. While valuable, in-situ observations are limited by uneven global distribution, quality inconsistencies, and high operational costs. Additionally, transforming in-situ observations into grid formats may distort the actual extent and timeline of flood events^19^. Satellite monitoring offers extensive coverage and helps fill observational gaps by tracking parameters such as soil moisture and flood extent, although it is limited by factors such as cloud cover and vegetation interference and requires ground validation^20^. Despite improvements in flood forecasting by integrating multiple data sources, challenges remain in detecting rapid flood events owing to the low temporal resolution and long latency of most satellite data. Nevertheless, operational flood forecasts have improved, supporting preparedness with enhanced short-to-seasonal range predictions^21^ facilitated by meteorological data and advanced models, such as LISFLOOD.

Water storage conditions are important in regions where antecedent conditions, rather than current rainfall, primarily trigger floods^6,22^. However, their roles are often assumed to be minimal and inadequately represented in flood analytical frameworks, thus underscoring a critical gap in flood analyses. For instance, in North American watersheds, groundwater-sustained river flow (i.e. baseflow) rather than soil moisture predominantly influences the magnitude of annual flooding events^23^. While routing models effectively simulate water levels in lakes and reservoirs and satellites offer insights into soil moisture, they represent only parts of the complex Total Water Storage (TWS) system. TWS encompasses a broader spectrum, including snow, surface water, soil moisture, and groundwater, which are important components of flood genesis and progression. In scenarios such as snowmelt-induced floods, or when runoff is mainly triggered by excess saturation runoff, relying solely on soil moisture data may prove inadequate owing to its limited sensitivity to flooding. This highlights the importance of adopting a holistic approach for flood analysis that encompasses the TWS system. The TWS-flood potential index^24^, informed by data from the Gravity Recovery and Climate Experiment (GRACE) and its Follow-On (GRACE (-FO)) missions^25,26^, offers a method for assessing flood risk by quantifying the changes in monthly TWS. This index calculates the storage deficit by contrasting the persistent peak storage with the previous month’s storage, providing an insight into the available storage capacity. As this deficit rises during dry periods and declines during wet periods, high precipitation events coinciding with low storage deficits result in an increase in flood risk^24^. The index was effective in predicting the 2011 Missouri River 500-year flood, yielding forecasts 5–11 months ahead of the event, and predicting large-scale summer floods in the U.S.^27^. However, the reliability of the flood potential index decreases when applied to unpredictable monsoonal flash floods. The monthly temporal resolution of GRACE (-FO) limits its capacity to predict short-term flood events. The current structure of this index treats each month's storage independently, neglecting the interconnected and sequential nature of TWS anomalies. By leveraging the sub-monthly tracks from GRACE (-FO) and accounting for long-term dependencies as Antecedent TWS (ATWS), the skills of GRACE (-FO) pre- and active-flood signals can be significantly improved.

Enhancing the temporal resolution of GRACE (-FO) results in a trade-off between temporal and spatial resolution with a higher temporal resolution, resulting in increased uncertainty in the spatial resolution of the GRACE data. The native spatial resolution of the monthly GRACE data is ~ 350 km. Previous research has generated 10-day solutions with spatial resolutions of 40 × 40 degree/order of spherical harmonics with an uncertainty of ~ 20 mm^28^. Daily and 10-day GRACE (-FO) solutions were obtained by compromising the spatial resolution. These efforts have incorporated ancillary data (e.g. hydrological modeling) and utilized autoregressive processes to overcome the challenges of limited satellite coverage and to enhance solution stability. A weighted 21-day sliding window was proposed for regularizing daily mass concentration (mascon) solutions^29^, and Kalman smoothers were applied to the daily spherical harmonics^30,31^. Although these solutions effectively capture high temporal variations in runoff during flood events^32^, their ability to estimate the flood extent is limited^32^. Incorporating a radial basis function enhances flood extent mapping^32^. A daily mascon solution based solely on GRACE land estimates has been proposed to recover meaningful sub-monthly signals over large spatial scales by iterating through the converged monthly solution^33^. Recently, line-of-sight inter-satellite ranging residuals have been used to track high-frequency mass changes at regional scales, proving to be effective during floods in Bangladesh in July 2020^34,35^.

Monthly aggregated GRACE-FO solutions are preferred for broad applications because they ensure complete global coverage of satellite tracking and reduce errors in mass change calculations. Nevertheless, the substantial latency of 40–60 days restricts their ability to monitor short-term events, particularly for signals such as floods^26^. When GRACE satellites operate outside deep orbits, they can achieve global coverage within 3–5 days, highlighting the potential for shorter data sampling and reduced latency (Fig. S1). Leveraging this opportunity, the Center for Space Research (CSR) at the University of Texas at Austin developed a new 5-day (5D) solutions based solely on GRACE (-FO) tracks with the same geodesic grid as the monthly solutions (~ 1-degree). Although 5D solutions inherently possess more noise and spatial biases, they can be applied to hydrological processes with large signals, such as floods^33^. The primary focus of this study was to assess whether GRACE (-FO) 5D solutions are more effective in detecting high-amplitude flood signals than traditional monthly solutions, particularly during various flood stages (pre-, active-, and post-flood). Guided by this objective, this study explored the following research questions:

1. How well do GRACE (-FO) 5D solutions represent pre-and active flood storage compared with traditional monthly solutions?
2. How does the 5D ATWS function as an early indicator of flood vulnerability when considering different flood mechanisms?
3. How rapidly do GRACE (-FO) 5D solutions monitor the storage responses to post-flood events and extreme rainfall?

In this study, we utilized GRACE (-FO) 5D solutions to develop an ATWS indicator for short- and long-term storage accumulation. We leveraged the DFO catalog, which reports 3272 flood events from 2002 to 2021, providing crucial details such as location, duration, and type ^15^. However, the DFO catalog is not comprehensive primarily because of its dependence on gauged basins and media coverage, which can overlook floods in remote areas and is subject to reporting delays^36,37^. We compared the ATWS derived from 5D solutions with the timing of reported flood events (i.e. onset and duration) to evaluate their effectiveness in flood detection and monitoring. The influence of ATWS on flood initiation and progression was quantified using the Precursor Coincidence Rate (PrCR). We then employed a hierarchical Bayesian model to assess the strength and uncertainty of the relationship between ATWS as a precursor and critical flood characteristics such as mechanisms, duration, extent, and severity. Additionally, we investigated post-flood and seasonal intense rainfall changes in the 5D TWS variations using a lagged Response Coincidence Rate (ReCR), which measures positive TWS changes following floods and intense rainfall, an essential aspect of water resource management. This study marks the initial phase in assessing the potential utility of GRACE (-FO) 5D solutions for operational flood monitoring.

## Results

We provide a concise overview of foundational concepts, gap-filling methodologies, and signal–noise evaluations for the current version of the 5D GRACE (-FO) solutions in the supplementary Information (SI) (Sects. S1–S3). This overview includes an analysis of the synoptic hydrological signal (less than 30 days) over land using 5D intervals, compared with daily data from GRACE ITSG 2018, atmospheric reanalysis, and land surface modeling.

### What are the primary causes of global floods from 2002 to 2021?

The primary cause of global flood events from 2002 to 2021 was heavy rainfall, which accounting for 76% of these events. Seasonal monsoonal rain and tropical cyclone followed, causing 10% and 9% of the floods, respectively. Snowmelt combined with heavy rain contributed to 4% of the events. Other causes, such as tsunamis, ice jam break-ups, and dam failures, are relatively rare, accounting for less than 1% of floods. We refer to this category as ‘other'. We focused on land floods rather than coastal floods because many other factors, such as storm surges, rather than ATWS, may be key factors in coastal flood generation. However, coastal floods were analyzed for post-flood impacts using the ReCR, which evaluates the probability of a wet 5D TWS period occurring after the flood.

We considered GRACE (-FO) 5D estimates over a 3-degree grid for each flood location based on GRACE (-FO) spatial resolution. Further categorization of floods based on their duration, severity, and magnitude is presented in SI, Sect. S4.

### How significantly do 5D GRACE solutions outperform monthly data in mapping pre-, active, and post-flood events?

**Figure 1:**
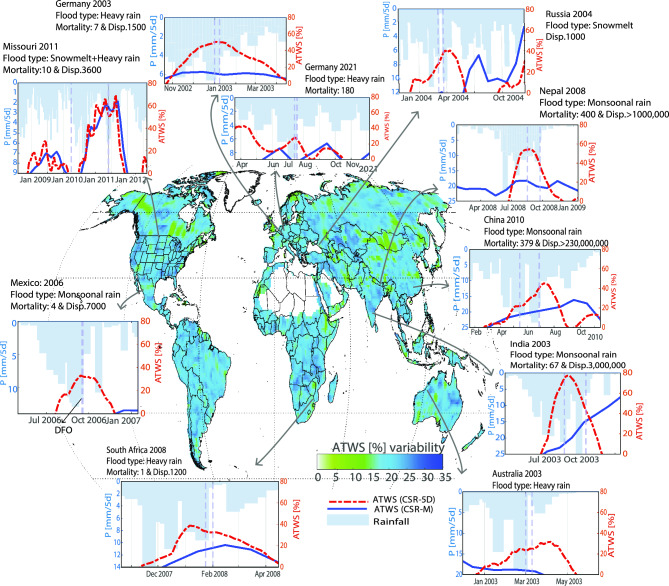
Pre-event Antecedent Total Water Storage (ATWS) signatures in 5D solutions. The base map shows the ATWS variability from April 17, 2002, to July 1st, 2022, with the climatological mean in Fig. S13. Subplots display ATWS wetness storage as a percentage over a 3-degree area surrounding flood locations for 5D and monthly mascon solutions. The monthly ATWS sums the current month and 50% of the previous month, whereas 5D solutions consider the current 5D period and the weighted prior six 5D intervals (6 × 5 days = 30 days, ~ 1 month equivalent). The equivalent precipitation (P) is shown. Vertical lines mark the start and end of a flood event, determined independently from the DFO catalog. The time series data for ATWS between 2002 and 2022 are shown in Fig. S14. Flood events are annotated with their location, year, flood mechanisms, and impact on livelihoods, including mortality and the number of displaced individuals. Plots were generated using MATLAB^39^.

In our analysis, we utilized the ATWS indicator of flood signals derived from both 5D and monthly GRACE (-FO) datasets, correlating them with flood timing (i.e., onset) and duration from the DFO database, to pinpoint storage anomalies preceding and during flood events (Figs. 1, S14). The ATWS calculations for each period represent the fraction of the relative wetness storage benchmarked against the historical TWS maxima from 2002 to 2022 (Fig. S12) and incorporating the cumulative storage from the previous month (“Methods” section).

The 5D solutions had much stronger pre-event signals than monthly signals for numerous floods. In the case of heavy rainfall and monsoonal floods, such as the 2003 Germany and 2008 Nepal floods, pre-event signals were only found in the 5D solutions and not in the monthly solutions (Fig. 1). Additional examples include the 2006 Mexico and 2021 Germany floods, which showed signals mostly in 5D solutions, with ATWS during these events ranging from 30 to 80% of the maximum wet storage. Snowmelt-driven events, exemplified by the 2004 flood in Russia, showed discernible pre-event storage increases in 5D data that were not captured by the monthly data. A notable snowmelt and heavy rain case was the prolonged storage buildup in the 2011 U.S. Missouri River event that occurred between March 2010 and June 2011. The 5D and monthly datasets showed a significant increase in ATWS, peaking at ~ 68% of the historical maximum before May 2011. The 5D solutions revealed greater ATWS variability, with storage increases preceding those in the monthly solutions. Although the TWS accumulation periods were similar, the monthly solutions proved less adept at detecting pre- and active flood signals compared to the 5D solutions. This discrepancy is likely due to the inherent data smoothing in the monthly solutions relative to the finer granularity of the 5D solutions.

**Figure 2:**
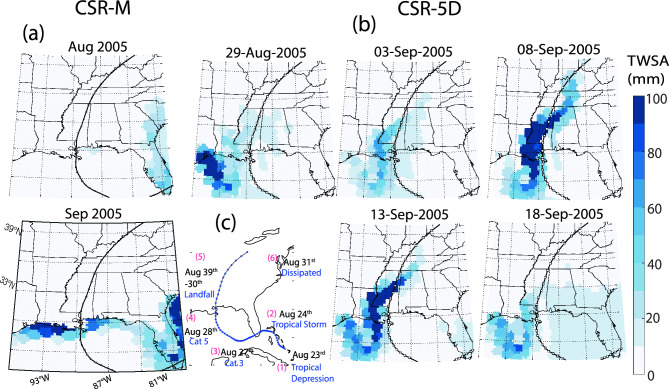
Utility of 5D in mapping atmospheric moisture transport events (i.e. Hurricane Karina). (**a**,**b**) Snapshots of the TWS change for both the monthly and 5D solutions after Hurricane Katrina, which made landfall between August 29th and 30th, 2005. (**c**) Originating as a low-pressure system in southeastern Bahamas on August 23, 2005, it quickly intensified, impacting southeast Florida, and later escalating to a category 5 hurricane, devastating the Louisiana and Mississippi Gulf Coast on August 29th and 30th, 2005, and dissipating on August 31st eastern Canada. The displayed TWSA is obtained by detrending and removing seasonal variations from the time series, with the August–September TWSA subtracted from that of July 2005. Only positive anomalies are depicted, highlighting the significant influence of hurricanes on water storage. Notably, monthly data exhibit no anomalies, vividly illustrating the initiation, evolution, and dissipation of anomalies along the hurricane trajectory in the 5D solution. All plots were generated using MATLAB^39^.

For post-flood signals, the 5D solutions also showed a clear advantage over the monthly solutions, particularly in capturing transient TWS changes following intense short-term events, as evidenced by Hurricane Katrina (Fig. 2)^38^. The TWS signature of this event was calculated by subtracting the detrended TWS in August and September from that in July 2005 for both the monthly and 5D solutions. While the monthly TWS solutions captured no significant post-hurricane anomalies, the 5D solutions distinctly revealed storage changes along the hurricane path, primarily impacting states such as Louisiana, Mississippi, Alabama, Tennessee, and Kentucky, particularly between September 8 and 13. By September 18, these pronounced TWS anomalies had diminished. The ability of 5D solutions to detect short-term fluctuations in TWS after Katrina highlights their value in providing detailed insights into post-event water-storage dynamics. Such fluctuations are often inherently smoothed in monthly averaged GRACE (-FO) data.

These findings show the superior capacity of 5D granularity for identifying both gradual and rapid TWS accumulation before and during active floods. This clearly distinguishes the usefulness of 5D data over monthly data in the early detection of flood signals, and in mapping and assessing post-flood conditions.

### How effective is the 5D ATWS as a flood precursor signal?

**Figure 3:**
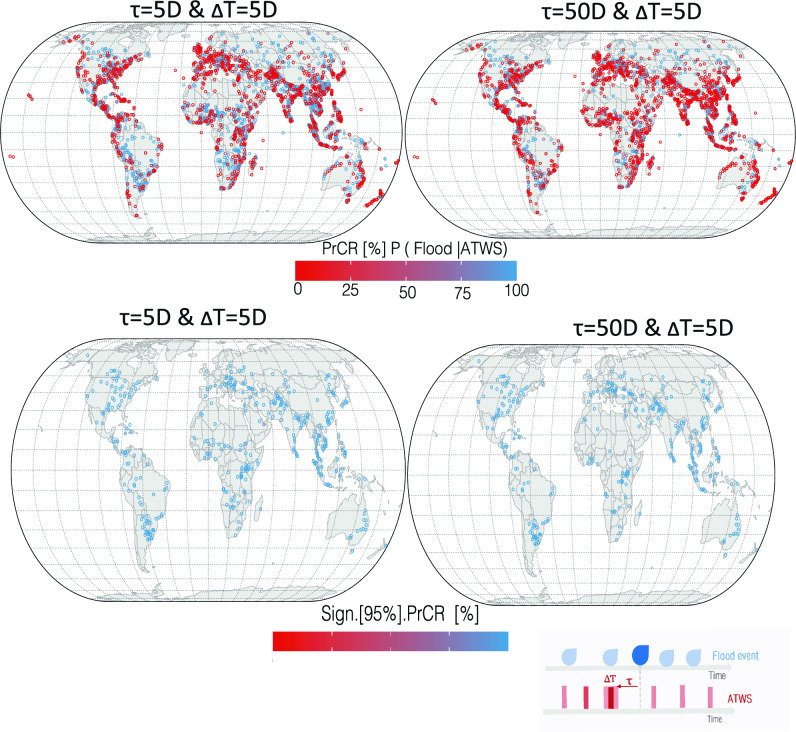
The precursor coincidence rate (PrCR) of the ATWS preceded or coinciding with 3272 flood events between 2002 and 2021 from the DFO catalog. Four maps illustrate the PrCR at temporal lags (τ) of 5 and 50 days and a tolerance window ($\Delta$T) prior to and concurrent with the event duration. The PrCR metric represents the proportion of flood days preceded by at least one 5D wet period with an ATWS > 0. A PrCR approaching zero denotes no overlap between the ATWS and flood days, whereas values equivalent to unity (100%) suggest complete overlap. The lower panel displays only those events for the PrCR rate with a 95% statistical significance. An empirical cumulative density function of PrCR, considering various τ and $\Delta$T, is depicted in Fig. S16. A snapshot of the PrCR concept is shown in the lower right of the figure. Plots were generated using R software^43^.

The role of the 5D ATWS as a precursor signal was quantified using the flood onset and duration of 3272 flood events based on the DFO catalog. The probability of a higher ATWS affecting flood onset and duration was evaluated using the PrCR (“Methods” section). PrCR conveys the fraction of flood days that coincide with or are preceded by at least one 5D period with an ATWS > 0. Consequently, it provides an indication of the strength of the statistical relationship between ATWS and the onset and progression of floods (Fig. 3).

The PrCR for the 3272 floods was quantified across various lead times prior to the flood onset and duration (Figs. 3, S16). On average, 80–85% of all DFO-reported flood events across various mechanisms were preceded by elevated ATWS, irrespective of the statistical significance of each elevated ATWS signal. Variations in the percentage of events with statistically significant PrCRs were influenced by the selected lag time between elevated ATWS and flood days, as well as by the tolerance window established for observing elevated ATWS. Notably, a longer tolerance window was correlated with a higher proportion of events that achieved statistical significance (Fig. S16). However, it is important to interpret these results within the broader context of their hydrological implications, rather than their mere statistical significance^40^. The probabilistic nature of ATWS influencing both the onset and duration of floods, as demonstrated through PrCR, provides a compelling narrative regarding the predictive utility of 5D ATWS, particularly when integrated with additional hydrometeorological variables. Conversely, the remaining 5–10% of floods, not preceded by elevated ATWS, were characterized by short-duration rainfall events lasting fewer than seven days. These events typically arose during periods of drought or marked rapid transitions from dry to wet conditions, often referred to as 'Whiplash events’^41^. Events such as tropical cyclones, atmospheric rivers, and significant shifts in synoptic weather patterns over periods ranging from days to weeks also played a role^42^ (Figs. S17, S18).

Heavy rainfall events showed an average PrCR of 0.58, indicating a moderate probability of occurring after and concurrently with a high ATWS. Notably, 75% of these overlaps had an increased PrCR between 0.66 and 0.75 when analyzed 22 days prior to flood occurrence. This suggests a strong link between ATWS as a predictive factor and the onset and development of floods from heavy rainfall, with an overlap probability of 66–75% over a 22-day lead time. Floods from monsoonal rain recorded an average PrCR of 0.63, assuming a 5D lag. Within this context, elevated ATWS was noticeable within 50 days prior to the floods, with coincidence rates fluctuating between 0.63 and 0.85. As lead times extended, the coincidence rates consistently stabilized between 0.70 and 0.75 within 30-day lead times. This trend emphasizes the sustained response of storage to seasonal rain, along with long-term accumulation, which culminates in soil saturation and subsequent runoff generation. The consistency of the elevated PrCRs underscores the significance of 5D sampling in capturing these dynamics. Floods initiated by snowmelt or rain-on-snow had an average PrCR of 0.69. For these scenarios, elevated ATWS was observed within 45-day lead time for approximately 70% of the time, with rates ranging from 0.50 to 0.85. Such higher variability underscores the influence of many factors, including antecedent snowpack conditions, melt rates, and man-made structures, such as dams and levees.

Although these probabilities illustrate the temporal association between elevated ATWS and flood onset within a specified window, they do not necessarily indicate a direct causative link. Nonetheless, the consistent temporal patterns indicated by the high PrCR values support a potential causal relationship between increased ATWS and subsequent flood events.

### To what extent does the precursor signal of the 5D ATWS vary with the flood characteristics?

**Figure 4:**
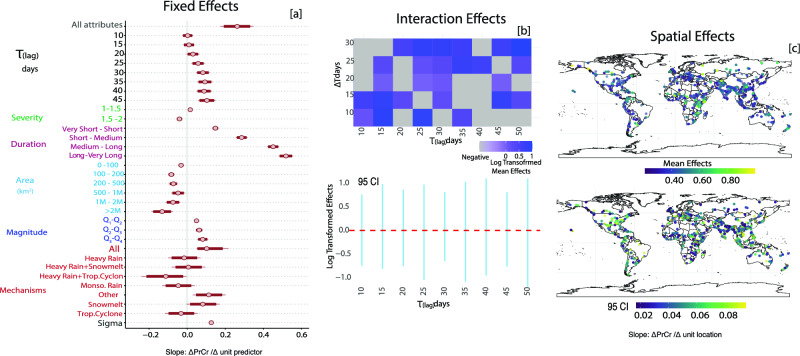
Relationship between flood attributes (i.e. duration, severity, magnitude, area, mechanism) and precursor coincidence rates: (**a**) Summary of fixed effects with mean and 95% credible interval (CI). The x-axis represents the slope of the PrCR, reflecting changes in the sign and magnitude of the PrCR relative to a unit change in flood attributes. Positive values indicate increases in PrCR (flood|ATWS) with each unit increase in the predictor, whereas negative values indicate decreases. Further discussion of flood attributes can be found in SI, sect. S4. (**b**) Interaction of lag or lead time (τ) and tolerance window (ΔT): Darker hues in the heatmap highlight positive PrCR impacts, whereas gray hues suggest limited or negative effects. The bar plot, marked with a 95% CI, reveals log-transformed effects at specific lags, emphasizing the values of shorter ΔT and τ of PrCR. (**c**) Spatial dependency: Portrays PrCR (flood|ATWS) spatial variance. Each event, marked by its mean and 95% CI signifies locales with an elevated PrCR, indicating the role of ATWS in projecting flood vulnerability. Events with positive values denote higher ATWS associations with flood locations (SI, S3, and S4). We used the same classification of flood mechanisms as reported by the DFO but categorized events such as breaches or glacial melts under ‘Other' because of their infrequent occurrence*.* Plots were generated using R software ^43^.

We applied a hierarchical Bayesian linear model to assess the relationship between ATWS PrCR and several key flood attributes, such as flood duration, severity, geographic location, spatial extent, magnitude, and generation mechanisms (Fig. 4a). The model, as detailed in the Methods section and SI, sections S4 and S5), effectively quantified the strength and uncertainty of the associations between PrCR and these attributes, focusing on cases where the PrCR was statistically significant with a p-value < 0.05.

Flood duration was strongly linked to higher ATWS PrCR, particularly in cases of extended flood periods. This pattern is also evident in floods caused by heavy rain, snowmelt, and rain-on-snow events. These conditions were associated with strong, positive  coefficients in the Bayesian hierarchical linear model. As characterized by persistent rainfall and consistent monsoonal patterns, these conditions frequently result in saturation excess runoff, where the ground becomes fully saturated. Under such conditions, ATWS plays an important role in flood development. The relationship between ATWS and flood duration shifted with rapid rainfall events such as tropical cyclones or brief monsoonal rains, resulting in weak and negative coefficients (Fig. 4a). The rapid onset of these floods shows that changes in ATWS may not provide sufficient lead time for effective forecasting, resulting in minimal or no overlap between flood days and ATWS (low PrCR). This dichotomy highlights the varying role of ATWS as a predictive tool, being more effective in longer-lasting floods, but of limited value for rapid-onset floods. Regarding flood magnitude, we found that its relationship with PrCR mirrors the patterns observed for the flood duration. This is intuitive because the flood magnitude is linked to both flood duration and area.

The Flood magnitude and ATWS showed a pronounced positive coefficient for regions such as East Africa, East Asia, and North America showed in contrast to Europe and South America (Fig. 4c). These variations reflect the distinct climatic and hydrological conditions in these regions, notably the influence of monsoon systems and snow-related flooding. Significantly longer lag times between ATWS and flood days show a strong positive association. This implies that considering a broader historical context, particularly through 5D ATWS storage accumulation, yields more accurate indicators of potential flooding. The varying strengths of the association across different lags reveal the intricate dynamics of flood genesis and progression, highlighting the variability in optimal predictive time frames. These findings align with the identified significant lead times of 22 days for heavy rainfall events and 45–50 days for snowmelt and monsoonal events. Overall, these results underscore the importance of integrating the temporal aspect in ATWS as a precursor signal prediction, emphasizing the necessity of customizing lag time analysis for specific flood mechanisms and characteristics.

The relationship between flood extent and PrCR is counterintuitive, as indicated by the negative coefficients in Fig. 4a. Larger flood areas do not always correlate with elevated ATWS before a flood, suggesting a complex interplay between geographical extent and flood development. Smaller areas with localized, intense rainfall can quickly elevate ATWS, but this does not always align with broader regional storage changes. In contrast, larger floods that cover multiple hydrological basins with diverse storage and drainage patterns present a more complicated relationship with ATWS.

In conclusion, ATWS is a reliable indicator of floods associated with saturation-excess runoff, particularly in regions with consistent heavy rain, snowmelt, and rain-on-snow conditions. Its effectiveness is reduced in situations of rapid onset and large-scale flooding. This highlights the potential of integrating ATWS with other prediction parameters to improve flood forecasting accuracy under varied hydrological and climatic conditions.

### How effective are 5D GRACE TWS solutions for monitoring changes in active and post-flood storage?

**Figure 5:**
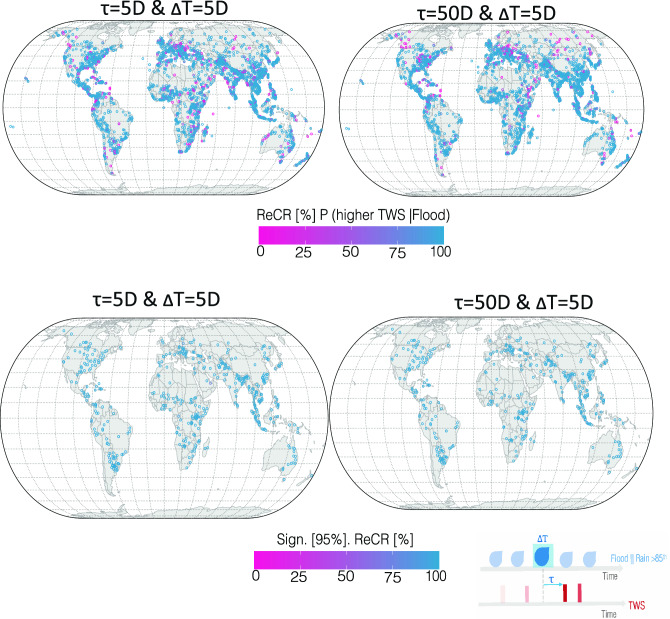
The response coincidence rate (ReCR) of 3272 flood events associated with detrended deseasonalized positive TWS levels between 2002 and 2021. Four maps display the ReCR at temporal lags (τ) of 5 and 50 days during and after flood events, with a tolerance window ($\Delta$T). The depicted percentages represent the fraction of positive detrended TWS 5D period that were preceded by at least one day of flooding. The lower panel displays only those events for the PrCR rate with a 95% statistical significance. An empirical cumulative density function of ReCR, considering various τ and $\Delta$ T is depicted in Fig. S19. A snapshot of the concept of ReCR is shown in the lower right of the figure. Plots were generated using R software^43^.

During and after a flood, a series of natural processes increases the TWS in the affected area. Floodwater initially saturates the soil and then overflows into rivers, reservoirs, and lakes. Excess water also percolates into the ground, effectively increasing groundwater levels and enhancing subsurface storage. The ability of 5D solutions to rapidly detect TWS changes during and after a flood is critical for efficient water resource management, which could enable prompt and informed responses in post-flood scenarios. We employed the ReCR metric to assess the effectiveness of 5D solutions in capturing and quantifying post-flood storage changes. This metric evaluates the likelihood of an active and post-flood period coinciding with positive TWSA. This is determination by calculating the proportion of TWS phases coinciding with or succeeding flood days. In this context, the temporal lag indicates the response time and dynamics of storage changes during and after floods (Fig. 5).

Our analysis reveals that, across all floods, ReCR values ranged from 0.35 to 0.71 within a 5–50-day post-flood response window. Significantly, the upper quartile of the ReCR values peaked at ~ 0.83, which is typically observed within 20–25 days. This shows that the most substantial increase in TWS was detectable within the 20–25-day period during and after a flood. Storage surge characteristics varied depending on the flood mechanism. For instance, floods caused by combined snowmelt and heavy rainfall induce sharper TWS increases within a 20-day window, as opposed to events such as Hurricane Katrina, which exhibit a distinct ReCR pattern averaging around 0.64 over a 5–50-day response time. These findings emphasize the effectiveness of the 5D solutions in rapidly detecting post-flood storage changes, which is important for efficient water resource management and post-flood assessments.

### How useful are the 5D GRACE TWS solutions for mapping storage changes after intense seasonal rainfall?

**Figure 6:**
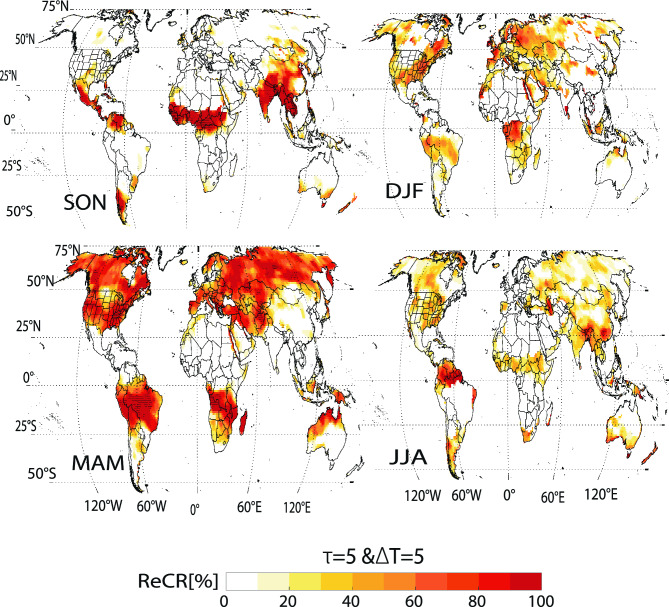
Response coincidence rate of intense rainfall (85th percentile) and an increase in synoptic TWS from 2004 to 2009 with no gaps in GRACE data. The four maps illustrate the ReCR at lags (τ) of 5 days for the four climate seasons. These percentages represent the fraction of synoptic TWS 5D period adjusted for the linear trend and both annual and semi-annual components, preceded by at least 5D period of intense rainfall. The varying patterns and percentages of the ReCR rate reflect the monthly seasonal extremes in climatology, as illustrated in Fig. S20. A sensitivity analysis using different lags and tolerance windows is presented in the results section. For further explanation, please refer to the methods section. Areas where the ReCR values are significant at 95% confidence interval are marked with black dots. *SON* September to November; *DJF* December to February; *MAM* March to May; *JJA* June to August. Plots were generated using MATLAB^39^.

We evaluated the effectiveness of 5D TWS solutions for detecting the storage signatures of intense rainfall events (> 85th percentile) using the ReCR on a grid scale (Fig. 6). To isolate these signatures accurately, TWS anomalies were filtered within a 15–60-day synoptic frequency after accounting for linear trends and annual and semiannual variations. Our analysis was confined to the 2004–2009 period to mitigate potential biases from incomplete GRACE (-FO) data records. The results reveal a pronounced spatial and temporal correlation between ReCR patterns and regional rainy seasons, effectively linking sub-monthly TWS anomalies with variations in seasonal precipitation (Fig. S21).

During the September–October-November (SON) season, high ReCR values ranging from 0.35 to 1.0, were found mostly in the monsoonal belt in regions such as Central Africa, India, Central America, and western Argentina. These high rates reflect an overlap between rainy days and high storage conditions. These regions align with the waning monsoon season in Central Africa and India, the initiation of the wet season in the western Pacific, later stages of the hurricane season in Central America, and the Caribbean, and the transitional phase between the two wet seasons in Argentina (Fig. S21)^44^. ReCR declines below 0.3 in south-central U.S., where regions with notably elevated ReCRs reflect overlaps between wet TWS 5D epochs and higher rainfall. Between December and February (DJF), increased ReCR ranging from  0.25 to 0.85 were observed in the Congo Basin and eastern U.S., indicating their respective rainy seasons^45,46^. Transitioning to March through May (MAM), the northern temperate zone displayed ReCR between 0.6 and 1.0, reflecting snowmelt and rain intensification^44^. June through August (JJA) showed elevated ReCR between 0.3 and 0.6 in areas such as sub-Saharan Africa and East Asia, whereas Europe and North America registered values below 0.4. These rates reflect the peak of the monsoon season or rainfall influenced by monsoons^27^as observed in East China and Korea^47^. The distinct seasonal and regional variations captured by the 5D solutions underscore their utility in capturing transient intense geophysical signals at a sub-monthly scale. A sensitivity analysis adjusting the response time between 5 and 50 days and changing the tolerance windows to 5–90 days (Fig. S20) indicates that the ReCR values remain consistent, even with extended lags. However, the most vital data are captured within a 5D lag, highlighting the value of 5D sampling. Altering the tolerance window to 15 days resulted in  approximately a 20% increase in the ReCR values across all climate seasons without affecting the statistical significance. The distinct seasonal and regional variations captured by the 5D solutions underscore their utility in capturing transient intense geophysical signals at a sub-monthly scale. These findings improve our understanding of the global hydrological cycle and its intricate interplay with weather and climate variations.

## Discussion

In this study, a novel 5D ATWS indicator was derived solely from the GRACE (-FO) 5D solutions. The 5D period represents the minimum time required to achieve global coverage. Distinctively, the 5D solutions function without ancillary monitoring or modeling data (Figs. S1–S9). The 5D ATWS data provide a window to capture both the short- and long-term dynamics of storage accumulations; thus, it serves as an early indicator of pre-flood storage conditions as shown by the PrCR and related flood vulnerability.

Compared to the monthly ATWS data, the 5D solutions show higher ATWS signal amplitudes and are more variable in certain types of floods, whereas ATWS signals are absent or less pronounced in the monthly data. This is particularly notable for floods resulting from heavy rain, monsoonal rain, and snowmelt (Fig. 1). The superiority of the 5D ATWS solutions is attributed to their finer temporal sampling, which captures both rapid and prolonged TWS changes, particularly those leading up to and during flood events. In contrast, monthly solutions, owing to their intrinsic smoothing processes, may miss such rapid dynamics. Additionally, the 5D TWS accurately tracked rapid storage shifts after floods, as observed after Hurricane Katrina, which was missing in the monthly TWS data, highlighting the limitations of the monthly data.

In our analysis of 3272 reported flood events, we identified 5D ATWS as a key predictor of flood initiation and progression. This assessment, derived using PrCR, revealed a significant relationship between flood characteristics and PrCR discerned through a Bayesian hierarchical linear model. This relationship is not limited to specific flood types; it spans diverse flood mechanisms such as rainfall, snowmelt, and rain-on-snow events. Uniformly elevated ATWS levels observed prior to these events, with lead times ranging from 15 to 50 days indicate the potential predictive capability of 5D ATWS. Moreover, the analysis sheds light on extended-duration floods, particularly those driven by continuous rainfall or monsoonal patterns; these floods are particularly evident in East Africa, East Asia, and North America (Fig. 4c). These findings emphasize the importance of pre-conditions not only in initiating floods but also in their prolonged progression, demonstrating the broad applicability of 5D-ATWS in both understanding and predicting flood dynamics. This aligns with previous studies highlighting the significant role of storage pre-conditions in flood initiation and progression. For example, experimental work has shown that storm runoff is largely influenced by pre-existing water storage rather than recent rainfall^48,49^. Furthermore, it has been found that river flow is primarily composed of water older than 3 months ^50^, and that groundwater, which often exceeds the soil moisture volumes in river dynamics, plays a critical role during storm events^49–51^. Integrating these processes, as shown by ATWS, offers valuable insights into the varying contributions of different processes to flood generation and dynamics. In practical terms, integrating 5D ATWS data with existing forecasting tools presents a promising opportunity to enhance flood-forecasting strategies. For instance, merging this data with global flood monitoring systems^52^, soil and water assessments^53^, and hydrological models^54^ could utilize the comprehensive estimates of the ATWS build-up provided by the 5D solutions. This integration is useful for differentiating between gradual increases in water storage due to snowmelt and sudden increases from rain events. Such differentiation improves the disaggregation of storage contributions from both surface and subsurface water components^55^.

Our findings reveal unexpected associations between PrCR and flood extent, underscoring the complexities of hydrological processes such as rainfall, runoff, and groundwater across various spatial scales. We found that floods with smaller extents have stronger associations with PrCR, showing a higher predictability of ATWS for flood initiation and progression. In contrast, floods with larger extents exhibited a stronger negative association with PrCR. These hydrological processes often display distinct non-stationary patterns that vary according to catchment size and characteristics^56,57^. In larger areas, hydrological variability is shaped by a mix of localized conditions within catchments and specific properties of aquifers, leading to divergent responses to similar rainfall events^56^. This indicates that the ATWS dynamics in larger catchments involve a more complex interplay of hydrological processes.

The 5D solutions, in their capacity to map post-flood water storage, provide a fresh perspective on the relationship between TWS dynamics and extreme hydroclimatic events. Our analysis underscores detectable TWS increases within a 20–25 day span post-flood, with variations tied to specific flood types. These findings highlight the potential of these 5D solutions for near-real-time mapping once operationalized. After extreme events, such as hurricanes and atmospheric rivers, 5D solutions can be valuable. Such events trigger pronounced changes in the hydrological dynamics, affecting flooding, groundwater recharge, and reservoir levels. Moreover, 5D solutions offer valuable insights, bridging gaps often omitted by monthly TWS solutions or other geophysical data with spatiotemporal constraints, including global positioning systems^58^, and soil moisture and groundwater monitoring networks. Seasonal evaluations further substantiate the granularity of the 5D solutions in capturing water storage shifts, particularly over regions of monsoonal rainfall. What stands out is the consistency of the response coincidence rates, even within varied tolerance windows (5–15 days), solidifies the reliability and precision of 5D sampling in capturing rapid storage responses following intense rainfall. This capability is paramount for timely and effective management of water resources. Integrating 5D TWSA sampling with additional data streams presents the potential for transformative impacts on water resource management.

In our investigation of GRACE (-FO) 5D solutions for flood analysis, we identified important areas for future research. The need for extensive error analysis and data refinement is critical for fully harnessing the operational potential of these 5D solutions. Our findings, supported by statistical evidence, demonstrate the capability of 5D solutions in flood applications and pave the way for future work that rigorously compares these solutions with other data sources, such as soil moisture estimates. This comparison is essential for determining how effectively 5D data can augment current flood forecasting methods. Addressing the spatial resolution limitations of GRACE (-FO) remains imperative because it is currently inadequate for detecting localized flood events. Moreover, the processing latency of monthly GRACE (-FO) data, which currently ranges from to 40–60 days, offers an opportunity for 5D solutions to enhance flood early warning, with the potential to reduce the latency to a mere 3–5 days in the operational mode. Ensuring the continuity of data, a cornerstone for reliable long-term flood monitoring, is a key focus for overcoming the current challenges posed by data gaps in the GRACE (-FO) time series.

Despite these challenges, 5D solutions adeptly harness the capabilities of GRACE (-FO) missions, delivering global updates every five days. Achieving this frequency with no ancillary modeling or observations marks a significant advancement in GRACE (-FO) technology. These developments aid in the monitoring and interpretation of rapid climate-related phenomena, providing a more dynamic and detailed perspective of Earth's hydrological and climatic systems.

## Methods

### GRACE (-FO) monthly and 5D solutions

The GRACE (-FO) satellite tracking data were processed to assess the Earth's surface mascon anomalies, presenting them as a uniform water layer within distinct mascons computed for each 5D interval. These solutions were created only from GRACE satellite tracking data, precluding reliance on external sources such as hydrological or statistical modeling. This is because most of the Earth is sampled within a 250 km swath at least once every 5 day, except when the satellite is in deep orbit. Mirroring the processing scheme of monthly solutions^59,60^, the 5D solutions were processed using standard procedures, including substitution of the C_20_ and C_30_ coefficients with satellite laser ranging estimates^61^, optimization of the geocenter motion C_21_ coefficients^62^, and glacial isostatic adjustment corrections via the ICE6G-D model^63^. These solutions use a geodesic grid model of the Earth's surface, comprising 40,962 cells (i.e. 40,950 hexagons and 12 pentagons), with coastal cells subdivided into land and ocean, resulting in 42,107 mascons. Each cell along the equator covered an area of ~ 12,000 km^2^ with an average distance of 120 km. The 5D data include the entire GRACE (-FO) record from April 2002 to July 2022. By incorporating a 'quick look' implementation, latency can be reduced to one day, and the solution period can be reduced to four days.

### Global flood catalog from Dartmouth Flood Observatory

The Dartmouth Flood Observatory at the University of Colorado provides a comprehensive catalog of large global flood events, drawing on diverse sources, such as remote sensing data (e.g. precipitation measurements and NASA Flood Alerts), river gauge data (e.g., USGS gauging stations), near-real-time remote sensing and modeling assimilations (e.g. the global flood monitoring system), storm tracking, and international news media reporting^15^. The dataset offers a multifaceted view of floods, capturing aspects such as the spatial extent, duration, severity, and impact. Smaller, localized floods or floods in uninhabited regions may not have been recorded in the DFO catalog.

### Precipitation

Daily precipitation data from the Global Precipitation Climatology Project (GPCP) were used to assess the rapid interaction between high rainfall and changes in storage. The GPCP provides a comprehensive and reliable global daily precipitation dataset at a scale of a 1° × 1° grid by combining satellite observations and rain gauge measurements^64,65^.

### GRACE (-FO) monthly and 5D total water storage: filling gaps

To address the data gaps within GRACE (-FO) and between GRACE and GRACE-FO for 5D solutions, we employed a Bayesian framework^66^ to model an individual time series. For 5D data, 283 solutions were missing between April 2002 and July 2022. We reconstructed 1477 solutions generated over both the existing and missing periods. Additional information on filling the GRACE data gaps is provided in SI (Sect. S3).

### GRACE (-FO) 5-day: Antecedent Total Water Storage

After filling the data gaps, ATWS was calculated by considering a weighted sum (W) of detrended TWS from the previous month (t-6) up to the present period (t) using Eq. (1),

1 $${ATWS}_{t}={TWS}_{t}+\sum_{i=t-6}^{t}{W}_{(i)}. {TWS}_{t-(6-i)}.$$

We employed a set of symmetric decay weights (i) (0.25, 0.39, 0.53, 0.67, 0.81 and 0.95), uniformly separated by an interval of 0.14, spanning a sequence from 0.25 to 0.98, to calculate the antecedent condition. This general approach is similar to that applied to the Antecedent Precipitation Index, which assigns greater weights to more recent events^67^. This approach recognizes the greater influence of recent events on the current state and gradually reducing the impact of past observations, which allows us to maintain their overall impact and accurately capture TWS inertia and memory effects. Subsequently, we normalized the resulting anomalies against the historical maxima of ATWS and converted the results into a binary series, assigning zero to negative anomalies and one to positive anomalies.

2 $${ATWS}_{norm}=\frac{{ATWS}_{t}}{{ATWS}_{max}}.$$

The monthly ATWS was calculated as the sum of the current month and 50% of the previous month to facilitate a fair comparison between the ATWS from 5D solutions and monthly solutions, and to enforce a balance between the current and past states of the TWS.

### Event coincidence analysis

Event coincidence analysis is a statistical technique that detects the temporal relationships between multiple events or time series^68^, such as elevated ATWS and flood onset, under the assumption that a higher ATWS coincides with or precedes floods. Coincidence analysis involves the calculation of two coincidence rates between events. The Precursor Coincidence Rate (PrCR) measures the frequency of increased ATWS occurring immediately before or during a flood event. The strength and directionality of the relationship between these event series were determined based on a lag window (τ) between the ATWS and flood onset, and a tolerance window (∆T) in which the ATWS could occur.

The other coincidence rate is the Response Coincidence Rate (ReCR), which assesses the proportion of flood periods or periods of intense rainfall that are followed by at least one wet TWS period, considering *the* lag and tolerance window; thus providing insights into the water storage response in flooded areas. Event coincidence analysis has been employed to establish the interrelations among a range of climate and environmental variables, including floods, drought-pluvial seesaws^69^, GRACE (-FO) monthly hydrological extremes^70^, and epidemic outbreaks^71^. The concept of coincidence is not symmetrical, and we used it in a two-way analysis. First, we assessed the relationship between flood onset and ATWS using the PrCR as a measure. In this scenario, the reference data were the flood onset and duration from the DFO catalog, which helped gauge the capacity of the ATWS to assess the flood initiation and progression. Second, we evaluated the usefulness of 5D TWS for post-flood storage using ReCR. Here, we reference TWS data to examine its ability to capture post-floods or intense rainfall exceeding the 85th percentile. Both rates are probabilities that vary from zero (indicating no coincidence between events) to one (signifying complete coincidence of events).

3 $$PrCR\left(\Delta T,\tau \right)=\frac{1}{{N}_{flood}}\sum_{i=1}^{{N}_{flood}}\Theta \left(\sum_{j=1}^{{N}_{{\varvec{A}}{\varvec{T}}{\varvec{W}}{\varvec{S}}}}{1}_{\left[0,\Delta T\right]} \left(\left({t}_{i}^{flood}-\tau \right)-{t}_{j}^{{\varvec{A}}TWS}\right)\right),$$

4 $$ReCR\left(\Delta T,\tau \right)=\frac{1}{{N}_{TWS}}\sum_{i=1}^{{N}_{TWS}}\Theta \left(\sum_{j=1}^{{N}_{flood}}{1}_{\left[0,\Delta T\right]} \left(\left({t}_{i}^{flood}-\tau \right)-{t}_{j}^{TWS}\right)\right),$$

where $\Theta (.)$ is the Heaviside function. For each flood event, flood duration (N) was obtained from the DFO catalog. We considered one climate season before the start of the flood and one climate season after its end to generate a time series of flood onset and corresponding ATWS and TWS changes. We performed a sensitivity analysis for different $\tau$ (5–50 days) and $\Delta T$ for each flood event at 1^∘^ ×1^∘^ grid scale. To further assess the strength of the statistical interrelationship between events, we conducted an analytical test using the Poisson approximation. Assuming that the event onsets are mutually independent and randomly distributed, we establish a null hypothesis (i.e. coincidence is randomly generated). We then calculated the statistical significance based on the p-value of the corresponding PrCR and ReCR rates for 1000 surrogate time series that had the same average time interval between the ATWS, TWS, and flood onset. Both the PrCR and ReCR for flood events were calculated for the ATWS after removing the harmonic components, such as the annual and semi-annual cycles, to ensure that the results reflect the intrinsic signal beyond those present in the monthly solutions. To capture the rapid response of the 5D TWS to intense seasonal rainfall (> 85th percentile), we utilized gap-filled data adjusted for linear trends and harmonic components (SI, Sect. S3). We then quantified the coincidence between precipitation (P) and TWS on a grid scale across different climate seasons.

### Bayesian hierarchical linear model

To determine the drivers of the statistically significant effect of the ATWS PrCR rate on flood onset, we integrated data on events from the DFO catalog into a Bayesian hierarchical linear model. We selected this model because of its ability to handle the hierarchical structure of flood characteristics.

5 $${\text{PrCR}}_{(\text{Flood}|\text{ATWS}}={\upbeta }_{0}+{\upbeta }_{1}\tau .\Delta T+{\upbeta }_{2}\text{Severity}+{\upbeta }_{3}\text{Duration}+{\upbeta }_{4}flood extent+{\upbeta }_{5}\text{Magnitude}+{\upsilon }_{\text{Mechanism}}+\in .$$

The model is structured with ${\upbeta }_{0}$ as the intercept, and ${\upbeta }_{1}$ to ${\upbeta }_{5}$ as the coefficients for the fixed effects corresponding to different flood-related predictors in determining the PrCR. These predictors include an interaction effect between τ and ΔT. The model incorporates random effects represented by ${\upsilon }_{\text{Mechanism}}$, which account for variations in PrCR associated with different flood mechanisms. The fixed effect coefficients, the random effects, and the residuals (denoted as ϵ) are all assumed to follow normal distributions. This assumption applies to the distribution of the coefficients and the random effects across different levels or groups defined by the flood mechanisms, not to the categorical variables themselves. The residuals ϵ are also assumed to be normally distributed, reflecting the variability in PrCR that is not explained by the model's fixed and random effects.

To capture the spatial dependence of the higher PrCR, we considered another spatial model, where the flood locations were modeled as:

6 $${\text{PrCR}}_{\text{Flood}|\text{ATWS}}=\propto +\mathcal{G}\mathcal{P}\left(\text{flood location}\right)+\in ,$$

where $\propto$ is a constant term, and GP (flood location) is a Gaussian process using an exponential quadratic kernel, modeling event spatial correlations via longitude and latitude. Additional details are provided in the SI (Sect. S5).

We employed the Stan No-U-Turn sampler to estimate model parameters, leveraging its state-of-the-art Markov Chain Monte Carlo (MCMC) capabilities intrinsic to the Stan language^72,73^. Four distinct MCMC chains were run, with each yielding 15,000 posterior samples per model. Subsequent inferences were derived by summarizing these samples using the mean and the 95% credible interval for each model parameter. Detailed methodologies are provided in section S5.

### Supplementary Information

Supplementary Information 1.

Supplementary Information 2.

## Data Availability

GRACE (-FO) monthly solutions are accessible through (GRACE/GRACE-FO—Gravity Recovery and Climate Experiment (utexas.edu). The 5D solutions are available from the second author upon request. Daily GPCP datasets can be sourced from Index of /data/global-precipitation-climatology-project-gpcp-daily/access (noaa.gov). The Dartmouth Flood Observatory hosts the global flood catalog, accessible at (Dartmouth Flood Observatory (colorado.edu)). Data associated with the results presented in this manuscript are provided as supplementary data and are available at: 10.18738/T8/6HKCGW.

## Code availability

The algorithms and software packages used in this analysis are detailed in the main text and supplementary materials.
