# Supplementary material for: Rapid mapping of global flood precursors and impacts using novel five-day GRACE solutions
Source: Sci Rep. 2024 Jun 15;14:13841. doi: 10.1038/s41598-024-64491-w (PMC11180122; doi:10.1038/s41598-024-64491-w)
Supplement: Supplementary file 1 — Supplementary Information 1. [file 41598_2024_64491_MOESM1_ESM.pdf]

Rapid Mapping of Global Flood Precursors and Impacts Using Novel Five-Day GRACE Solutions

Ashraf Rateb[*1], Himanshu Save[2], Alexander Y. Sun[1], Bridget R. Scanlon[1]

[1]Bureau of Economic Geology, Jackson School of Geosciences, The University of Texas at Austin, Austin, Texas 78758, United States
[2]Center for Space Research, University of Texas at Austin, Austin, Texas 78759, United States
*Corresponding author: Ashraf Rateb (ashraf.rateb@beg.utexas.edu)

Five sections
23 Figures.
29 Pages

Section 1: GRACE/GRACE-FO Five-Day Solutions Data Generation

The solutions for the Gravity Recovery and Climate Experiment and its Follow-On (GRACE-(-FO) missions are typically computed monthly using a span of 30 or 31 days. This design guarantees sufficient global satellite coverage and enhances the accuracy of the global inversion of the gravity field. However, the Earth's surface is usually covered within a 250-km swath at least once every five days, except during deep repeat phases. This observation suggests that five-day (5D) solutions based solely on satellite tracks are viable.

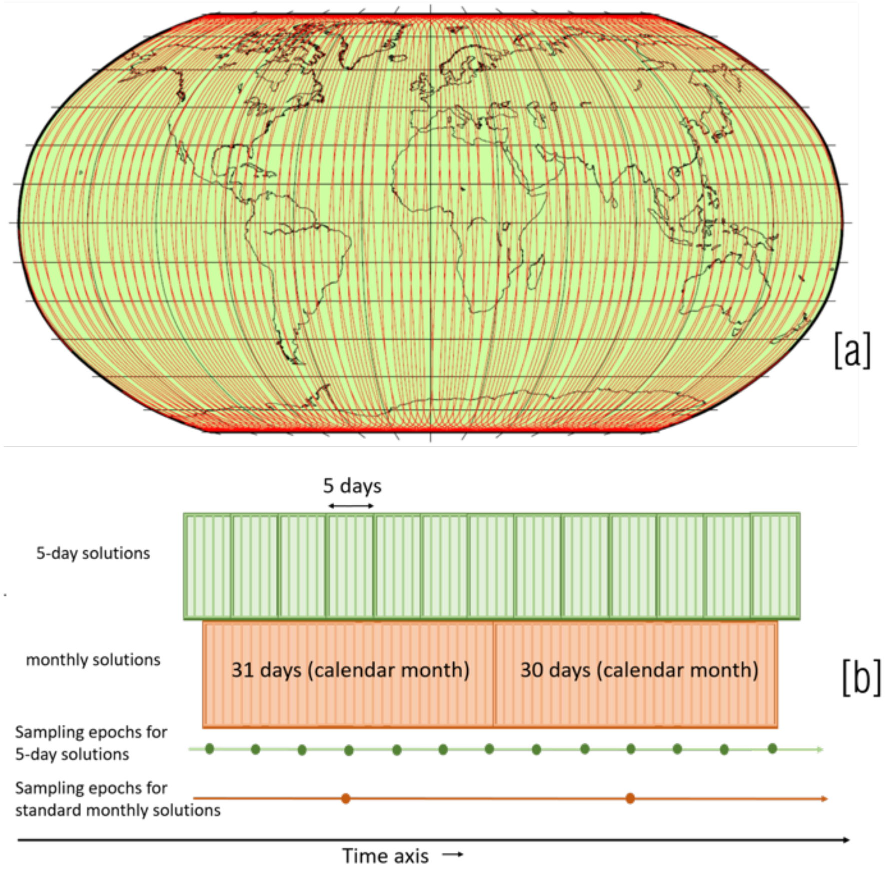

Fig. S1. [a] Global coverage achieved by GRACE/GRACE-FO tracks over a 5D period, which underpins the University of Texas at Austin Center for Space Research (CSR) mass concentration (mascon) of 5D solutions. [b] Sampling difference between traditional monthly solutions (30/31 days) and newer 5D solution epochs.

By leveraging this high frequency, we can detect more detailed shifts in global mass anomalies. These 5D solutions were developed using the RL06.2 monthly mascon processing scheme at CSR and are derived solely from GRACE/GRACE-FO tracks, without reliance on external data or modeling.

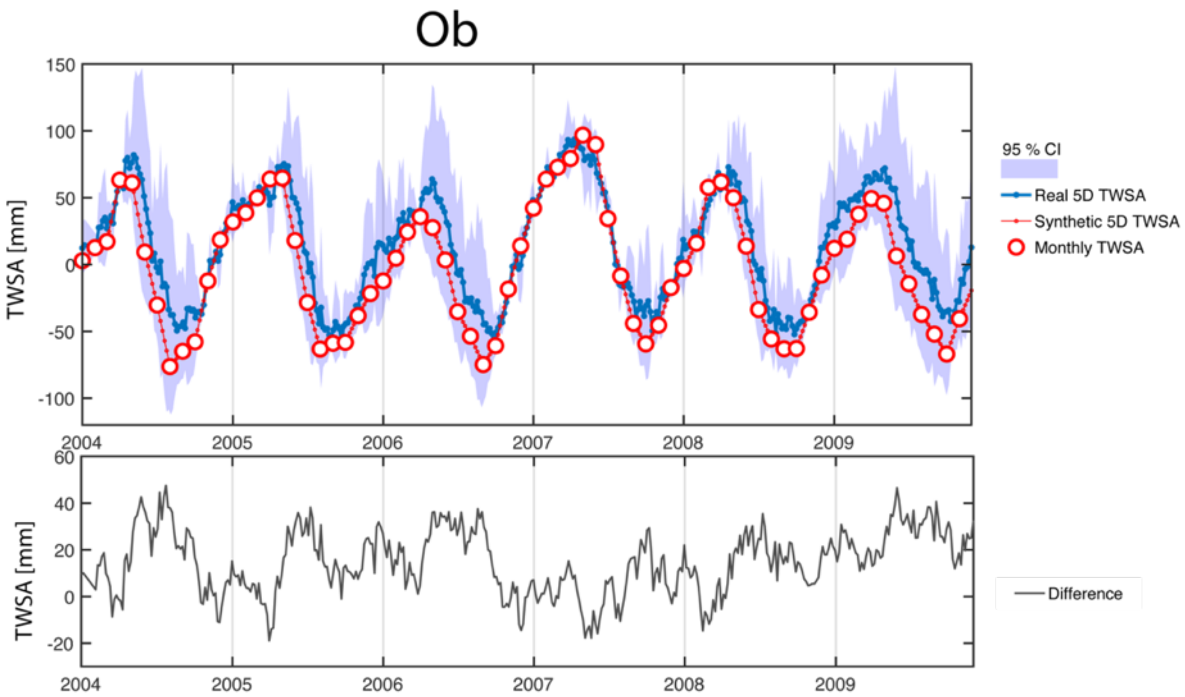

Fig. S2. **Upper panel**: Total Water Storage Anomaly (TWSA) in the Ob Basin from 2004 to 2009. The real 5D solutions, shown in blue, capture the short-term fluctuations in the TWSA, including the noises of 5D solutions track averaging. The red curve illustrates the synthetic 5D TWSA solutions derived from the interpolated monthly TWSA and offering smoother data representation. The actual monthly data are denoted by red circles. The shaded blue region surrounding the real 5D solution curve shows a 95% confidence interval based on the difference between the synthetic and real 5D solution data, with wider regions showing greater uncertainty or variability.
Lower panel: the difference between the real and synthetic 5D solutions TWSA, underscoring variabilities stemming from real 5D solution-track averaging. Substantial deviations from zero on the black line show periods where real 5D solutions data diverge from its synthetic counterpart, showing noise or pronounced transient geophysical signals in the real 5D solutions solution.

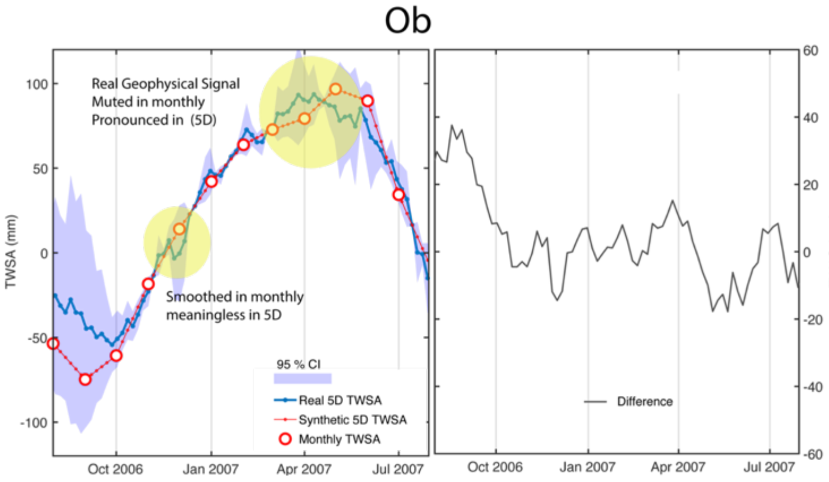

Fig S3. Mirroring Figure S2 but narrows its focus to the period between August 2006 and August 2007, emphasizing the variability and interpretation of both monthly and 5D GRACE solutions. The circle marking December 2006 on the real 5D solutions suggests minimal additional insights from these solutions and indicates inherent noise in short-term measurements. Conversely, the circles in March and April 2007 highlight the 5D solutions' portrayal of pronounced TWS amplitude during, which appears smoothed in the monthly solutions. This contrast underscores the additional variability of real geophysical signals captured by the real 5D solutions.

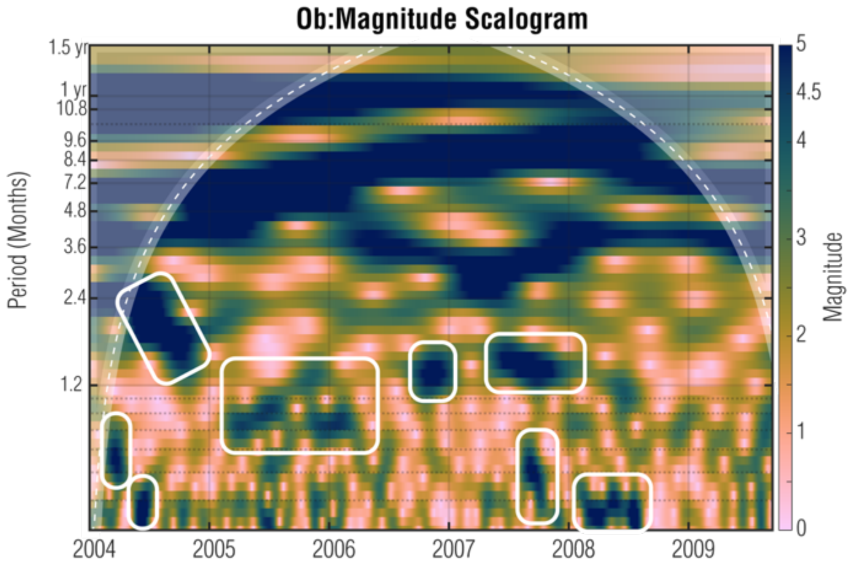

Fig. S4. The scalogram illustrates the continuous wavelet transform of the differences between real and synthetic 5D solutions (i.e., the lower panel of **Fig. S2)**. This wavelet analysis, with its ability to capture both localized and broad-scale changes, accentuates the significance of high-amplitude regions.
The vertical axis denotes the period in months, spanning one month to 1.5 years, while the color bar signifies magnitude levels. The white dashed curve highlights zones with a noteworthy spectral power. Dominant periodic signals are clear as horizontal bands. Areas outlined in white pinpoint specific time-frequency regions, suggesting potential anomalies or unique submonthly events in the real 5D solutions TWSA. Such pronounced signals may indicate hydrological events such as intense rainfall or rapid snowmelt. These signals may serve as precursors to major hydrological events that can be muted or overlooked in monthly solutions.

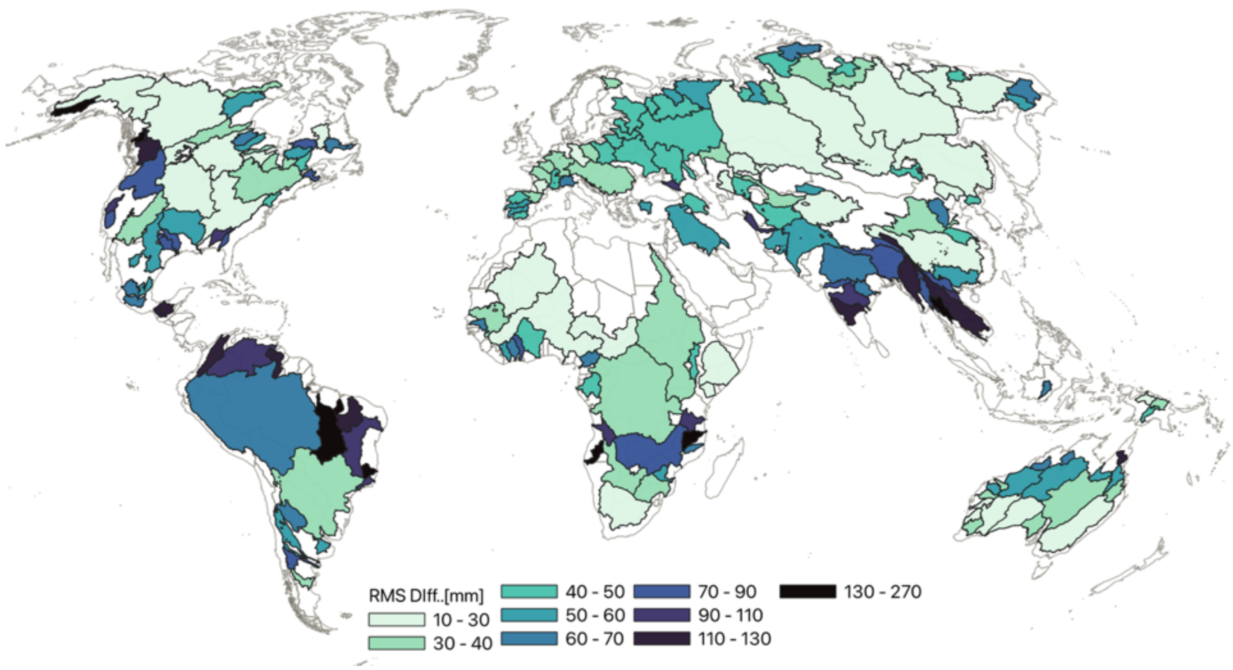

Fig. S5. Spatial distribution of root-mean-square error (RMSE) values for real 5D GRACE solutions data compared to synthetic 5D solutions across 186 global hydrological basins, showing the absolute difference in millimeters. The color gradation, from light green to dark blue and black, represents increasing RMSE values ranging from 10 mm to over 270 mm. Lower RMSE values in lighter shades suggest closer agreement between the two datasets, while higher values in darker shades identify regions with greater discrepancies. These absolute error magnitudes are essential for quantifying the residuals in the TWSA when transitioning from monthly to 5D solutions averaging, reflecting the increased noise levels associated with more frequent observations.

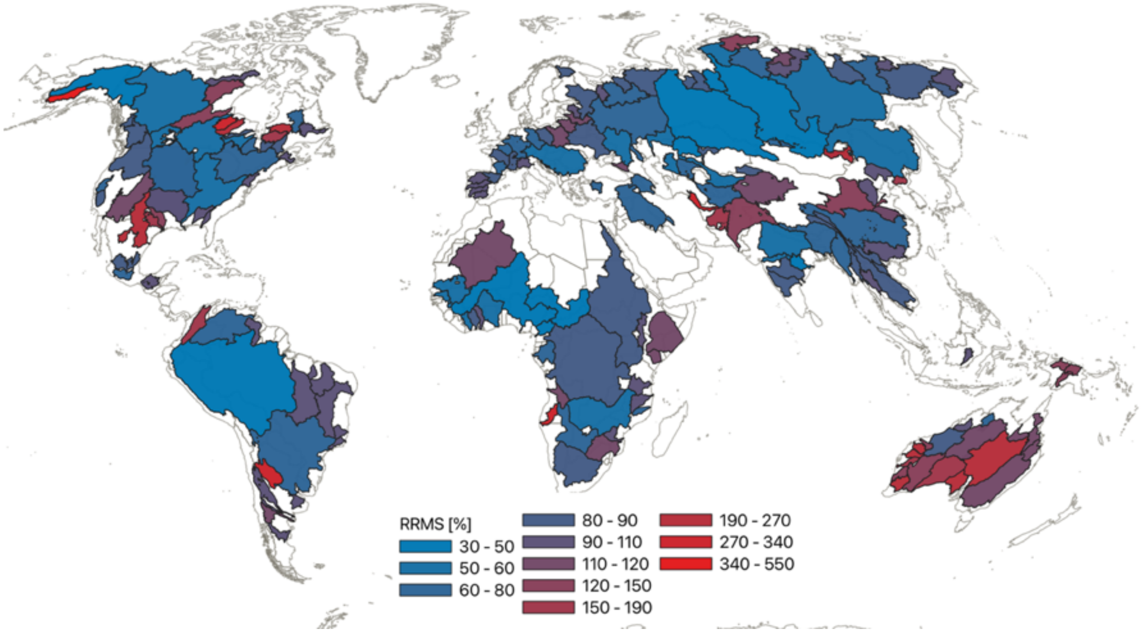

Fig. S6. Global distribution of Relative RMSE (RRMSE) in real 5D solutions GRACE Data: RRMSE, calculated as the normalized differences between the real and synthetic 5D solutions data. The color scale ranged from lighter to darker shades, representing lower to higher RRMSE values. Lighter areas show a minimal deviation from the synthetic (i.e., monthly averaging) baseline, suggesting lower variability. In contrast, darker regions highlight significant differences, implying greater variability because of the amplified signal and noise in the 5D solutions averaging process.

Section 2: Validation of the Sub-monthly Synoptic Hydrological Signal in 5D Solutions

S2.1. Data and Analysis

We assessed the significance of the 5D solutions submonthly signal for hydrological applications considering the rapid changes in retained or released water. Our analysis drew upon another GRACE daily solution (i.e., ITSG-2018) [1], Hydrological Land Surface Discharge Model (LSDM) [2], atmospheric reanalysis data such as the Modern-Era Retrospective Analysis for Research and Applications (MERRA) [3], and the fifth generation ECMWF atmospheric reanalysis of the global climate (ERA-5) [4]. We considered four years of free-gap data spanning from 2005 to 2008 for the estimates of the TWSA and rate of change in the climatological TWSA (dS/dt).

Changes in the TWS encompass various continental storages, such as groundwater (GW), surface water (SW), soil moisture (SM), snow water equivalent (SnW), ice, and canopy. These components can be simulated using the LSDM model. The rate of change (dS/dt), which can be viewed as residuals of the water balance over a 5D scale, can be derived from data sources such as MERRA-2 and ERA-5. The 5D solutions were provided at a resolution of 0.25° × 0.25°. In comparison, both the ITSG-2018 and LSDM offer daily estimates on a one-grid scale. Reanalysis datasets are accessible at 3-hour to daily intervals, spanning grid scales from 0.1° × 0.1° to 0.5° × 0.6°.

To ensure consistency in the comparison, all datasets were re-sampled to a 1-grid and 5D interval.

$$\mathrm{TWS}_{\mathrm{GRACE}\,(-\mathrm{FO})} = \mathrm{GWS} + \mathrm{SWS} + \mathrm{SM} + \mathrm{SnW} \quad (1)$$

$$\frac{\mathrm{dS}}{\mathrm{dt}} = \frac{\mathrm{TWSA}_{(t+1)} - \mathrm{TWSA}_{t-1}}{2\mathrm{dt}} = \mathrm{P} - \mathrm{ET} - \mathrm{RO} \quad (2)$$

where P is the total precipitation,
ET is the total evapotranspiration,
and RO is the total runoff.

For a comparative analysis with the LSDM, we detrended the TWS from 5D solutions at the 1-degree grid scale, segmented its frequency bands, and isolated signals with frequencies ranging from **11 days to a month using the inverse Morelt wavelet transform** [5]. The rate of water balance change from 5D solutions (dS/dt) was calculated using central numerical differences, incorporating a weighted filter, denoted as

$$\mathrm{W}_{(t)} = 0.25\mathrm{W}_{(t-1)} + 0.5\mathrm{W}_{(t)} + 0.25\mathrm{W}_{(t+1)} \ (3)$$

where W is P, ET, or RO.

It is important to acknowledge the limitations of this comparison. The components from equation (2) are indicative rather than exhaustive due to the inherent limitations of reanalysis data, such as the omission or inadequate representation of certain physical processes such as groundwater recharge, irrigation, runoff, and potential evapotranspiration. Residuals from the reanalysis can be equated to surface soil moisture changes, whereas 5D solutions (dS/dt) encapsulate the rate change of all water storage types.

For TWS, our comparison was solely between the 5D solutions and the LSDM-simulated model. An illustration highlighting the extracted submonthly signal and the time series from the four datasets for dS/dt across ten major hydrological basins is presented in Fig. S7.

Our evaluation employed five statistical metrics to gauge phase alignment, variability, amplitude, and probability distribution similarity: correlation coefficients (R), RMS, standard deviation ratios, unbiased RMS difference (uRMSD), and Kullback–Leibler divergence (KLD) (equations, 4-6).

$$r = \frac{\sum(x_i - \bar{x})(y_i - \bar{y})}{\sqrt{\sum(x_i - \bar{x})^2 \sum(y_i - \bar{y})^2}} \quad (4)$$

$$\mathrm{RMS} = \sqrt{\frac{1}{n}\sum x_i^2} \qquad (5)$$

$$\mathrm{KL}(P||Q) = \int p(x)\log\left(\frac{p_i(x)}{q_i(x)}\right) \qquad (6)$$

For r x_i, y_i is the variable (x, y) at time i, $\bar{x}$, $\bar{y}$ are the averages of x, y. KL is a normalized symmetric score between 0 and 1 based on information theory. KL reflects the expectations of divergence between the model (P) (here, the 5D solutions) and referenced data (Q: here, the other four data independently) if we replace one with another. A score of zero indicates an identical probability distribution between the two datasets, and a score of one shows the two distributions are maximally different. We used the Jensen-Shannon divergence symmetric version p (P||Q)=p(Q||P) [6,7].

S2.2. Comparative Assessment of Sub-monthly Hydrological Signals: Phase Agreement, Amplitude, Variability, and Distribution Similarities Across 5D Solutions and Global Datasets (ITSG, LSDM, MERRA-2, ERA-5

In terms of phase agreement for TWS and (dS/dt), 5D solutions demonstrate strong correlations with other datasets, including LSDM for TWS and ITSG, MERRA-2, and ERA-5 for dS/dt. Specifically, they align closely with ITSG solutions across most land areas (Fig. S8), exhibiting a correlation exceeding 0.4 at over 60% of the land grid points. Additionally, notable agreements with LSDM are observed, with correlations greater than 0.2. The consistent alignment observed between the 5D solutions, and ITSG-2018 solutions is expected, as both derive from GRACE (-FO) missions, albeit through distinct data generation methodologies. Specifically, 5D solutions capture gravity changes across most of Earth's surface within 250-km swath every five days, while ITSG utilizes a recursive Kalman filter applied to spherical harmonics up to degree and order 40, coupled with an AR (3) process based on the LSDM dynamic model to address the limited track coverage of GRACE (-FO) within a 24-hour window. Consequently, varying degrees of alignment with LSDM and reanalysis data are anticipated due to inherent physical limitations, biases, and uncertainties in these model-based datasets. This observation aligns with findings from previous research, such as by Eicker, et al. [8]., who noted discrepancies between ERA-5 variants and ITSG-2018 solutions within the same signal bandwidth.

The uRMSD metric reveals a consistent pattern of signal variability. For more than 80% of the land grids, the amplitude difference between the 5D solutions and reanalysis data ranges from 10 to 13 mm per 5D solutions. ITSG shows a closer amplitude alignment with the 5D solutions. For TWS, the difference between the 5D solutions and LSDM is approximately 15 mm for over 80% of all land grids (Figs. S8, S9). The relative variability ratio indicates that 5D solutions exhibit greater variability in this signal band compared to other datasets. Compared with ITSG, this ratio ranges from 1 to 2, except in regions such as the Caspian Sea and Great Lakes, where 5D solutions record higher values. The disparity in variability is more pronounced in desert regions of Northern Africa, the Arabian Peninsula, and Iran when comparing LSDM and MERRA-2. Outside these desert areas, the variability patterns closely resemble those of ITSG. The most significant divergence in the relative variability ratio is observed with ERA-5, where in many regions, including South America, West and Central Africa, the Sahara, Northern India, and the Western U.S., the 5D solutions display variability over six times that of ERA-5.

When evaluating the KLD results, which assess the similarities and differences in probability distributions between the 5D solutions and the four other datasets, ITSG stands out with the smallest divergence, exhibiting an approximate score of 0.06 for over 80% of all grid points. In contrast, the other datasets (LSDM, MERRA-2, and ERA-5) display scores ranging from 0.13 to 0.15 for a similar percentage of grid points, with the LSDM metric being slightly higher. Notably, areas with smaller KLD values for ITSG and LSDM align with regions where the variability ratio between these datasets and 5D solutions is low, such as North Africa and Central Asia. An intriguing aspect arises in the analysis of ERA-5: in regions with low variability ratios, the differences in probability distributions between ERA-5 and 5D solutions are

minimal. However, in humid regions like the tropics and Eastern U.S., despite high variability ratios, the KLD values also trend higher, suggesting that ERA-5, despite having reduced variance in these areas, aligns more closely with the average values of 5D solutions.

The pronounced amplitudes and variability of the submonthly signal across tropical and humid regions, such as the eastern U.S., correlate with the dynamic hydrological processes prevalent in these areas. Frequent and rapid shifts in water flux, exacerbated by precipitation levels that exceed evaporation for much of the year, lead to significant runoff. These rapid fluctuations are driven by a variety of processes including oceanic-atmospheric interactions, monsoon patterns, land-sea interactions, intense rainforest convection, and local energy cycles [9-11]. While similar processes occur in humid temperate zones outside the tropics, they manifest with less intensity. In contrast, areas like North Africa and the Arabian Peninsula, with minimal hydrological activity, show subdued hydrological changes at the submonthly scale due to their sparse hydrological dynamics[12].

The detection of consistent structural variations across the GRACE solutions, LSDM, and reanalysis datasets in submonthly frequency bands bolsters our confidence in the validity and relevance of the 5D solutions-derived signals. The ability to identify rapid hydrological patterns at this scale represents a significant advancement in our understanding of, and ability to track, phenomena such as floods and flash droughts, which occur on daily to weekly scales. This capability exploits the full potential of the GRACE (-FO) missions with respect to temporal resolutions, making submonthly TWS data crucial for early warning systems and risk assessments. While our analysis focuses primarily on the complexities of submonthly signal variability, it underscores the necessity for in-depth research to elucidate the specific causes behind these shifts in TWS and exploring the full capability of the 5D solutions. Despite certain limitations, such as increased noise and compromised spatial resolution in the GRACE (-FO) missions, 5D solutions offer a unique opportunity to improve LSM data assimilation and atmospheric reanalysis through incorporating the state of the TWS at submonthly scales. These capabilities are crucial for accurately simulating extreme events and assessing hydrological responses to land-water energy exchanges.

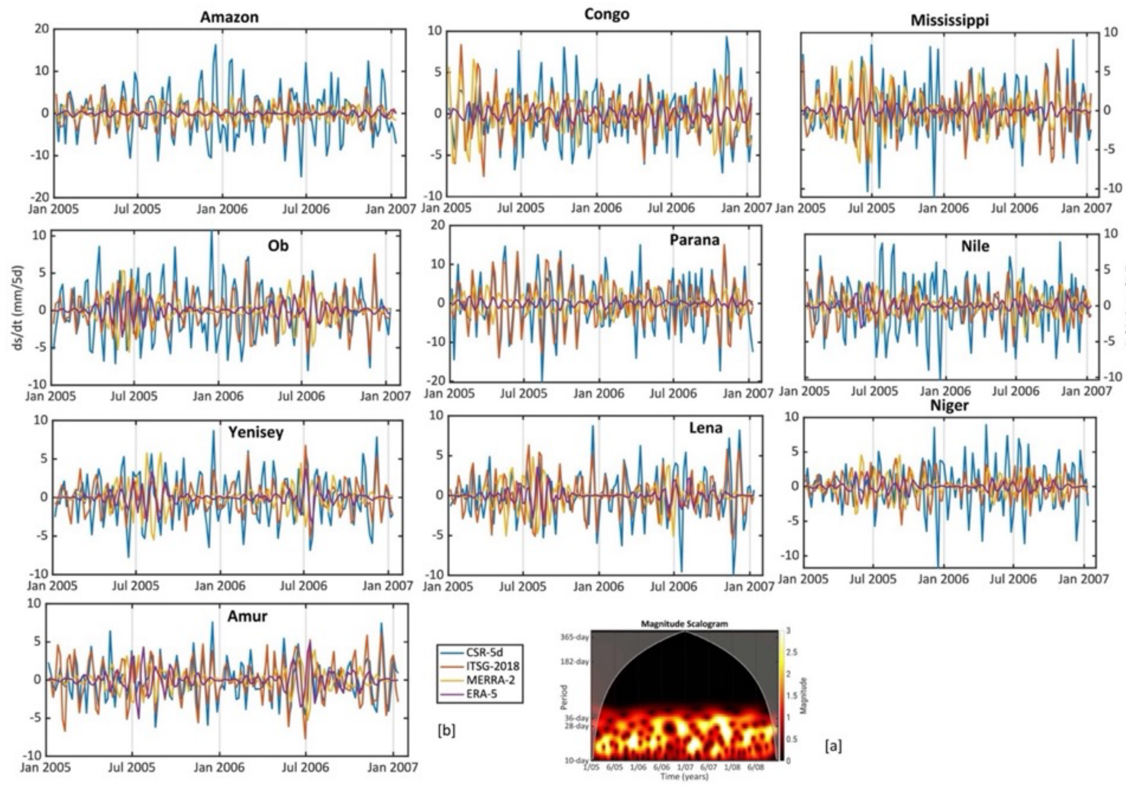

Fig. S7. [a] Scalogram of the sub-monthly signal (dS/dt) derived 5D solutions for the Amur basins. [b] Time series of the dS/dt) signal, with a frequency band of 11-day to 1-month, derived from 5D solutions, ITSG, MERRA-2, and ERA-5 data for the ten major hydrological basins.

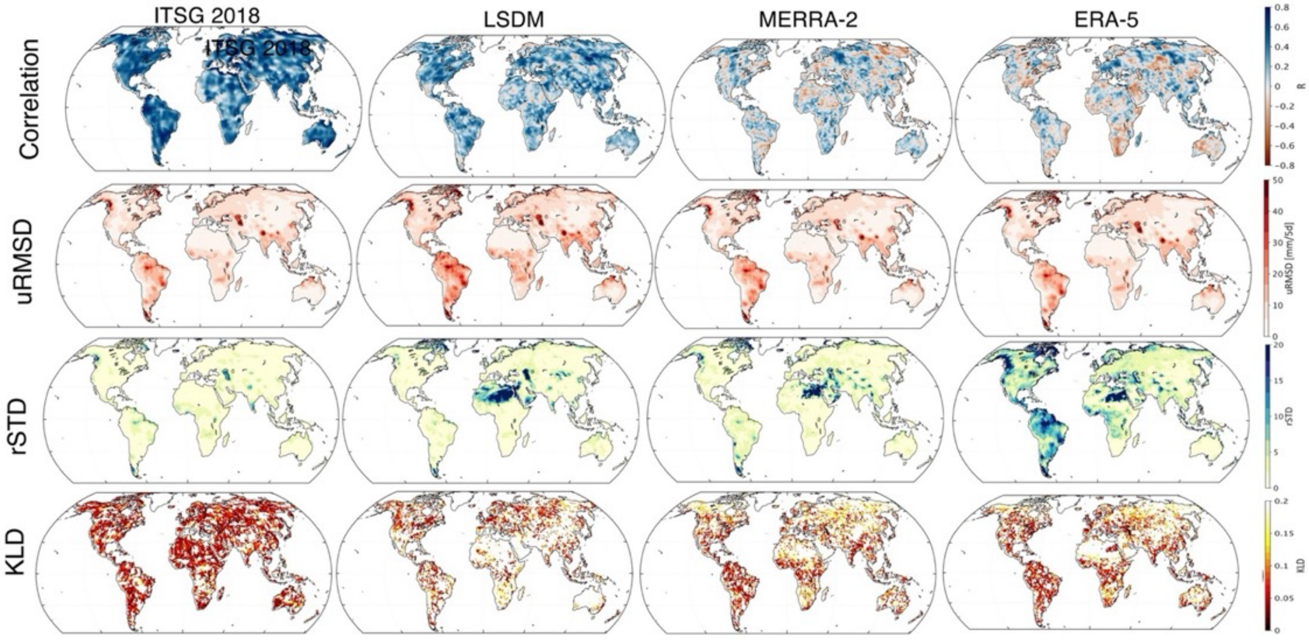

Fig. S8. Comparative visualizations of 5D solutions and other datasets including ITSG, LSDM, MERRA-2, and ERA-5. Top row: Unbiased central root mean square. Second row: Root mean square differences. Third row: Ratio of standard deviations. Fourth row: Kullback-Leibler divergence.

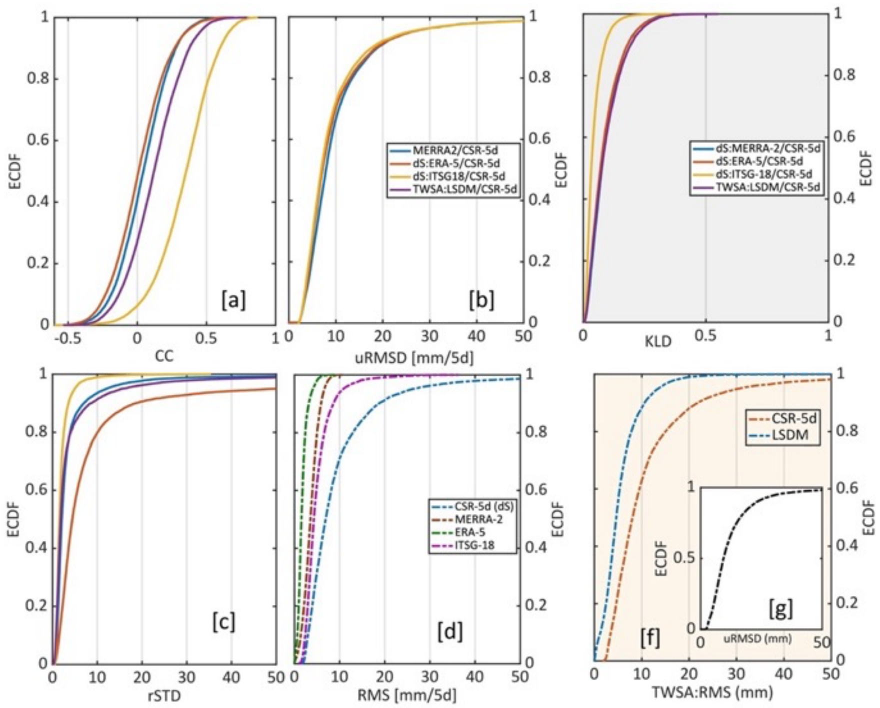

Fig. S9. Empirical cumulative density functions showcasing four major tests: correlation coefficient distributions (a), unbiased central root-mean-square differences (b), ratios of standard deviations (c), root-mean-square variability (d), Kullback-Leibler divergence (e), root-mean-square plots of TWSA derived from CSR and LSDM (f), and unbiased central root-mean-square difference between 5D solutions and LSDM TWSA (g).

Section 3. Filling the Gaps within and between GRACE (-FO) Missions for Monthly and 5D Solutions

The need to bridge gaps in GRACE/GRACE-FO data arises because these gaps significantly undermine the quality of our long-term observations and predictions. Any discontinuity in the data series may lead to skewed results, potentially causing misinterpretations. Therefore, a comprehensive and continuous dataset is required. The missing solutions within and between the GRACE and GRACE-FO missions for the monthly and 5D solutions were filled using state-space models (SSMs) in a Bayesian framework[13]. SSMs within a Bayesian framework offer several key advantages over other methodologies. First, SSMs exhibit remarkable flexibility by accommodating various time-series structures, enhancing their adaptability to unique dataset conditions. This flexibility is evident as they account for long-term trends, annual and semi-annual effects, and irregular fluctuations in the data. Such an approach allows for detailed decomposition of GRACE (-FO) data into individual signal components, facilitating a better understanding of the impact of missing data on each component[13]. Second, parameter estimations within a Bayesian framework allow the incorporation of prior knowledge and derive the uncertainties associated with signal components[14].

Following Rateb, et al. [15], GRACE (-FO) (Y) signal can be decomposed into the following

$$\mathrm{Y}_t^{\mathrm{GRACE\ (-FO)}} = \mu_t + \delta_t + \gamma_{jt}\cos(\lambda_j) + \gamma_{jt}^{*}\sin(\lambda_j) + \epsilon_{t,} \quad t = 1, \dots . n. \quad (7)$$

where μ_t is the intercept, δ_t is the slope, $\gamma_{jt}, \gamma_{jt}^{*}$, are the annual or semi-annual amplitudes for cos, and sin, respectively, ϵ_t is the residual, t is the time, n is the number of observations.

$\lambda_j = 2\pi j/S$, where S is the frequency of the annual as 12-month (73 epoch) or semi-annual 6-month (36 epoch) for monthly and (5D) solutions.

In a probabilistic framework, components are stochastic and vary randomly. Their coefficients also drift according to a random walk.

For intercepts

$$\mu_{t+1} = \mu_t + \delta_t + \epsilon_{t,} \qquad \epsilon_t \sim T_{v_\mu}(0, \sigma_\mu), \qquad (8)$$

For slope

$$\delta_{t+1} = \delta_t + \eta_t \qquad \eta_t \sim T_{v_\delta}(0, \sigma_\delta), \quad (9)$$

For annual or semi annual

$$\gamma_{jt+1} = \gamma_{jt}\,cos(\lambda_j) + \gamma_{jt}^{*}\,sin(\lambda_j) + \epsilon_{0t} \qquad \epsilon_{0t} \sim N(0, \sigma) \quad (10)$$

$$\gamma_{jt+1}^{*} = \gamma_{jt}^{*}\,cos(\lambda_j) - \gamma_{jt}\,sin(\lambda_j) + \epsilon_1. \qquad (11)$$

Where η_t is an independent random variable with zero mean, $and\ \sigma_\eta$ standard deviation. Both $\epsilon_t\ and\ \eta_t$ are mutually independent and from δ_t. $v_\mu$is the level tail thickness, v_δ denotes the slope-tail thickness.

When $\eta_t = 0$, the trend is exactly deterministic linear, except for a noise model.

ϵ_0, ϵ_1 are independent random variables with identical variances.

ε is the residual shared among all the components.

For semi-annual signal we repeat the trigonometric part in equation (7)

The trend component in this equation captures both the linearity and interannual decadal fluctuations in the GRACE (-FO) data.

In a Bayesian framework, the GRACE (-FO) data and the temporal component parameters (θ) can be described:

$$P(\theta|D) = \frac{P(D|\theta) \times P(\theta)}{\sum P(D|\theta) \times P(\theta)} \qquad (`12)$$

where $P(D|\theta)$ denotes the probability of observing GRACE (-FO) data (likelihood), given the temporal component parameter θ; $P(\theta)$ is the prior distribution of the parameters, $P(\theta|D)$ is the posterior distribution of θ. The denominator is the sum of the likelihoods given by priors.

Determining the posterior distribution necessitates knowledge of the explicit forms of both the prior and likelihood, a hurdle that can be overcome through sampling-based methods like Markov Chain Monte Carlo (MCMC). We used the No-U-Turn Sampling (NUTS) technique across four Markov chains. Each chain underwent a 'burn-in' phase of 5000 iterations to establish a stable distribution. This phase was succeeded by an additional 5000 iterations to generate the posterior distribution of each component. To ensure the robustness of our model, we implemented a rigorous approach combining cross-validation for time series with MCMC convergence diagnostics. We started by employing a diagnostic test of convergence, specifically the $\hat{R}$ statistic, and aimed for a value of ≤1.05.

By evaluating the variance within and across chains, with $\hat{R}$ values approaching one showing superior convergence, this test ensured the reliability of the component parameter convergence. Subsequently, we calculated the means and associated 95% credibility intervals of the distributions for all components, interpreting these means as the overall mean reconstructed signals. This step was followed by integrating the residual component with the existing observations to preserve the variability of the original data. To further validate the efficacy of our model and its decomposition capability, we employed a time series-specific cross-validation method. This involved dividing our data into ten folds, maintaining the chronological integrity of the series, and using a rolling or expanding window approach for the training and testing sets. An example illustrating the application of our method for filling the monthly and 5D solutions is shown in Fig. S10 using the Mississippi River Basin as a case study. We assessed the performance of the model using statistical tests, root-mean-square error, mean absolute deviations, and the coefficient of determination, as shown in Fig. S11.

Section S4. Deriving Flood Parameters

Within the DFO catalog, flood severity is categorized into three distinct classes, each of which is indicative of the impact level.

- **Class 1 - Significant Flood Events:** These floods result in considerable damage to infrastructure and agriculture, lead to loss of life, and occur with a reported frequency of once every 1-2 decades.
- **Class 1.5 - Very Large Events:** These floods transpire once every two decades to a century and affect geographic regions spanning over 5,000 km^2.
- **Class 2 - Extreme Flood Events:** Representing the pinnacle of severity, these floods are deemed rare, with an estimated occurrence interval exceeding a century. Their rarity underscores their extreme nature and the potential for widespread devastation.

Within the DFO catalog, each flood event was defined by its start and end dates, providing a specific duration. We converted the duration data into a numeric format and then categorized it into distinct classes: Very Short (0-10 days), Short (10-20 days), Medium (20-40 days), Long (40-60 days), and Very Long (>60 days). Furthermore, the catalog provides information on the geographic extent of floods, quantified in square kilometers. These extents, derived from globally curated news reports, depict regions affected by flooding. While these polygons offer a visual representation of affected areas, it is pivotal to note that they symbolize regions influenced by floods rather than the exact inundated zones. The magnitude of the flood was determined using a combined metric based on three key parameters: duration, severity, and area.

$$\text{Flood magnitude} = \log(\text{Duration} * \text{Severity} * \text{Area}). \quad (13)$$

For ease of analysis, flood magnitudes were categorized into quartiles (Q1 to Q4) representing the 1st through 4th quartiles of the flood magnitude distribution. We used the same classification of flood mechanisms as reported by the DFO but categorized events such as breaches or glacial melts under 'Other' because of their infrequent occurrence.

Section 5. Determinants of the Significant PrCR

S5.1. Model 1: Fixed effects and interaction effects of PrCR (ATWS|flood)

To identify the key factors influencing the statistically significant PrCR rate of Antecedent TWS (ATWS) on flood timing, we incorporated data on events from the DFO catalog into a Bayesian hierarchical framework as

$$\text{PrCR}_{(\text{Flood|ATWS}} = \beta_0 + \beta_1\tau.\Delta T + \beta_2\text{Severity} + \beta_3\text{Duration} + \beta_4 flood\ extent + \beta_5\text{Magnitude} + \upsilon_{\text{Mechanism}} + \in \quad (14)$$

β_0 is the intercept, $\beta_0 \sim\mathcal{N}(\mu_{\beta_0}, \sigma^2_{\beta_0})$
β_1 for the interaction of the lag (τ) between higher ATWs and flood onset and ΔT, the window of the higher ATWS could occur, $\beta_1 \sim\mathcal{N}(\mu_{\beta_1}, \sigma^2_{\beta_1})$, β_2 for flood severity, $\beta_2 \sim\mathcal{N}(\mu_{\beta_2}, \sigma^2_{\beta_2})$,
β_3 for flood duration, $\beta_3 \sim\mathcal{N}(\mu_{\beta_3}, \sigma^2_{\beta_3})$, β_4 for flood extent,
$\beta_4 \sim\mathcal{N}(\mu_{\beta_4}, \sigma^2_{\beta_4})$, β_5 for flood magnitude,
$\beta_5 \sim\mathcal{N}(\mu_{\beta_5}, \sigma^2_{\beta_5})$, $\upsilon_{\text{Mechanism}}$ for the random effects of flood mechanism $\mathcal{N}(o, \sigma^2)$,
and $\in$ is the model errors term $\mathcal{N}\ (o, \sigma^2)$

We deliberately choose non-informative or flat priors for our model. Despite the keen interest in the interplay between the ATWS and flood parameters, the current understanding remains nebulous. Flat priors ensure our model is primarily shaped by data and is free from external biases. They also allow a comprehensive exploration of the parameter space, especially when the relationships among variables are uncertain, preventing hasty conclusions.

S5.2. Model 2: Spatial effects using Gaussian Processes Regression

To assess the spatial predictability of ATWS, we developed a spatial model using a Gaussian Process (GP) with a rational quadratic kernel.

$$\text{PrCR}_{\text{Flood|ATWS}} = \propto + \mathcal{GP}(\text{flood location}) + \in \quad (15)$$

$$\text{PrCR}_{\text{Flood|ATWS}} \sim \mathcal{N}(o, \sigma^2)$$

The GP regression models nonlinear relationships using a set of prior functions. The key idea is to assume a distribution over functions, which is determined by the kernel function. The kernel function represents the covariance structure between data points, which defines the relationships in the function space [16].

Here, we used a rational quadratic kernel as

$$\mathcal{GP} \sim K(x_a, x_b) = \sigma_f^2 exp\left(-\frac{\|x_a - x_b\|^2}{2l^2}\right) \quad (16)$$

Where $K(x_a, x_b)$ denotes the covariance function between floods location,
σ_f^2 symbolizes the maximum function variance, which adheres to a student's t-distribution with three degrees of freedom, a mean of 0, and a scale parameter of 2.5. This describes the inherent variability in the data. Student's t-distribution is a heavy-tailed distribution that provides robustness against outliers and captures a wider range of potential variabilities. Its use suggests that we are allowing for the possibility of occasional large deviations from the mean.
ℓ is the length scale of GP, distributed according to inv-Gamma (1.5,0.05). This parameter defines the rate at which the covariance of the function decays with distance and captures the spectrum of spatial

structures. A smaller length scale implies that the function values can change more rapidly, leading to a "wiggly" realization, whereas a larger length scale implies smoother functions. The inv-Gamma distribution's shape and scale parameters are set to 1.5 and 0.05, respectively, to allow for flexibility in modeling a wide range of spatial structures.

We used the advanced MCMC capabilities of Stan[17,18] NUTS to determine the model parameters. Four MCMC chains were deployed, producing 15,000 posterior samples for each model parameter. These samples were then analyzed to derive inferences, with the mean and 95% credible intervals for every parameter serving as primary summaries.

S5.3. Models Diagnostics

To discern the reliability and precision of our Bayesian models, we employed a multifaceted diagnostic approach to examine key metrics that offer insights into the model behavior and convergence quality of our Monte Carlo approximations. The results of these diagnostic tests are illustrated in Figs S22 and S23. The Monte Carlo Standard Error MCSE gauges the uncertainty associated with the Monte Carlo approximation, signaling the closeness of our posterior samples to the true posterior distribution. The effective sample size provides a measure of the number of independent draws from the posterior distribution, accounting for the inherent autocorrelation within a chain. A satisfactory ESS ensures that the model adequately explores the parameter space and is the key to stable estimates. The $\hat{R}$ statistic serves as a convergence diagnostic, juxtaposing the behavior of individual chains to the collective behavior of all chains. When all the chains achieved equilibrium, they adequately explored the distribution.

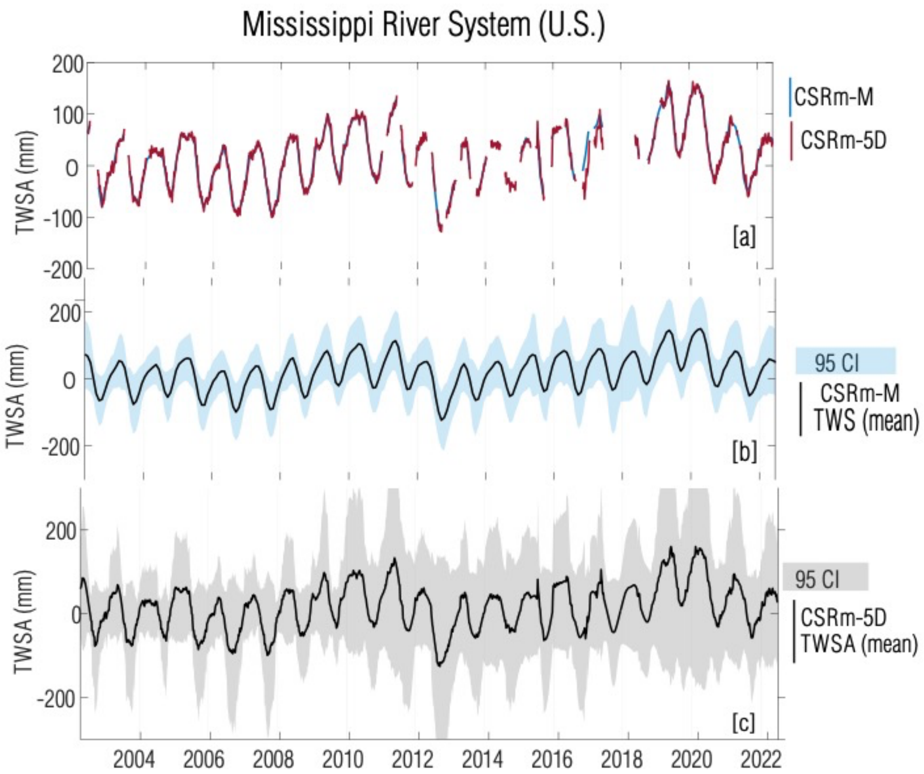

Fig. S10. Gap filling of the CSR mascon monthly and 5D solutions with GRACE(FO) data. [a] Time series of the TWSA in the Mississippi River system (U.S.), featuring data gaps for both monthly and 5D solutions. [b] reconstructed data for monthly solutions, presented as the mean of the distribution along with its 95% credible interval. [c] reconstructed data for 5D solutions, presented as the mean of the distribution with its corresponding 95% credible interval.

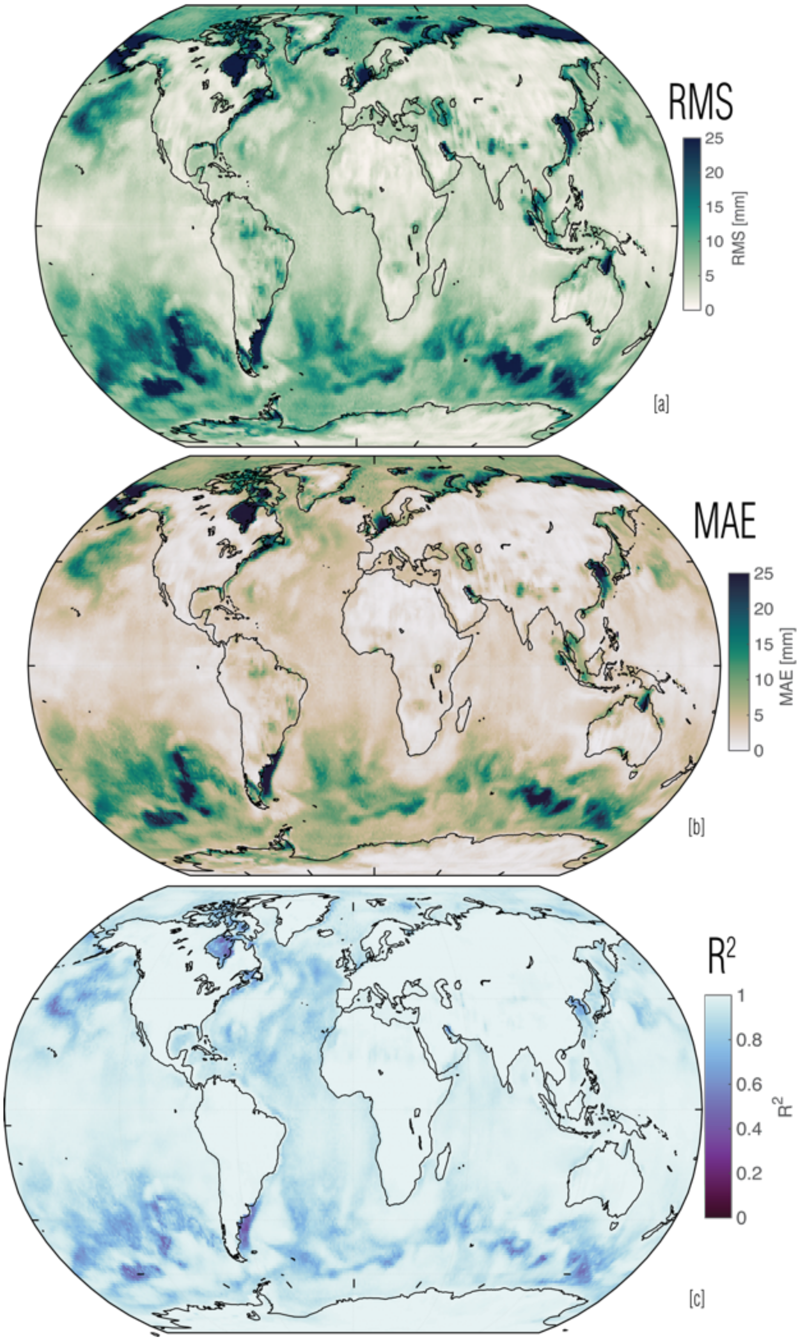

Fig. S11. Cross-validation results of SMM for 5D solutions data gap imputation. The evaluation metrics includee, root-mean-square error (RMSE), mean absolute deviation (MAE), and R-squared.
The figure highlights the performance of the model across various tests, underscoring the accuracy and reliability of the Bayesian approach in reconstructing the TWS for 5D solutions.

.

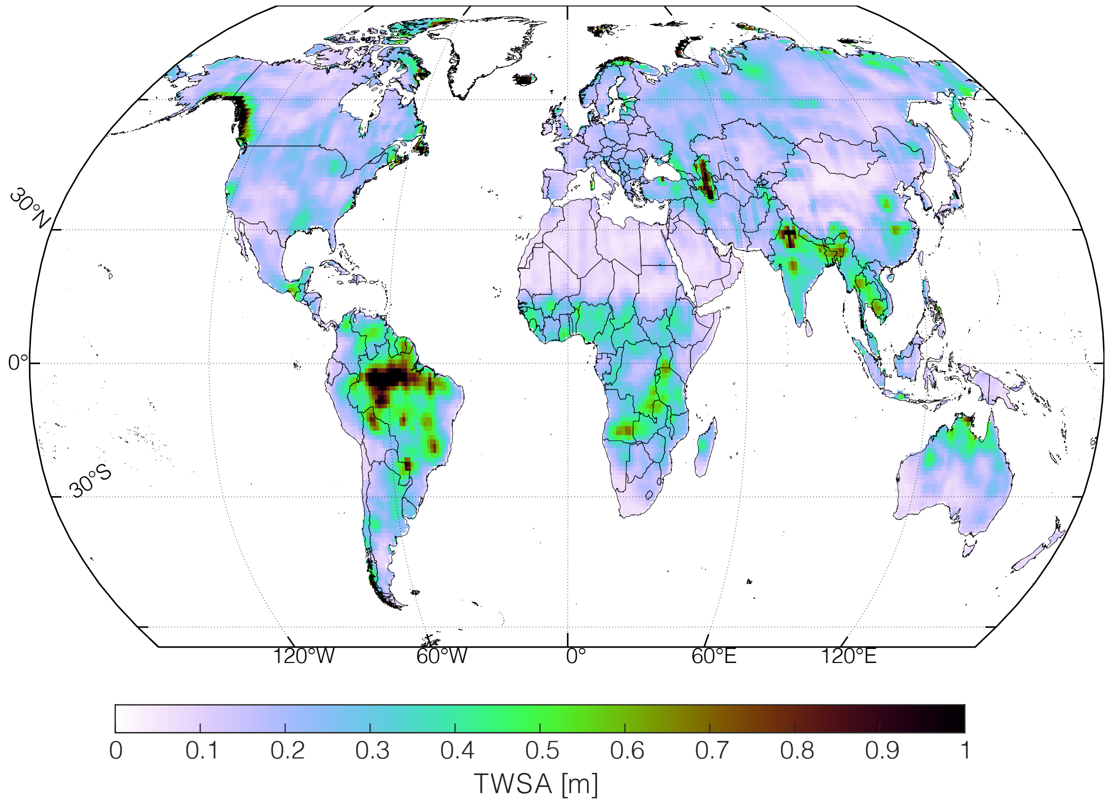

Fig. S12. Historical maxima of TWSA between April 17, 2002, and July 1, 2021, based on new 5D mascon solutions.

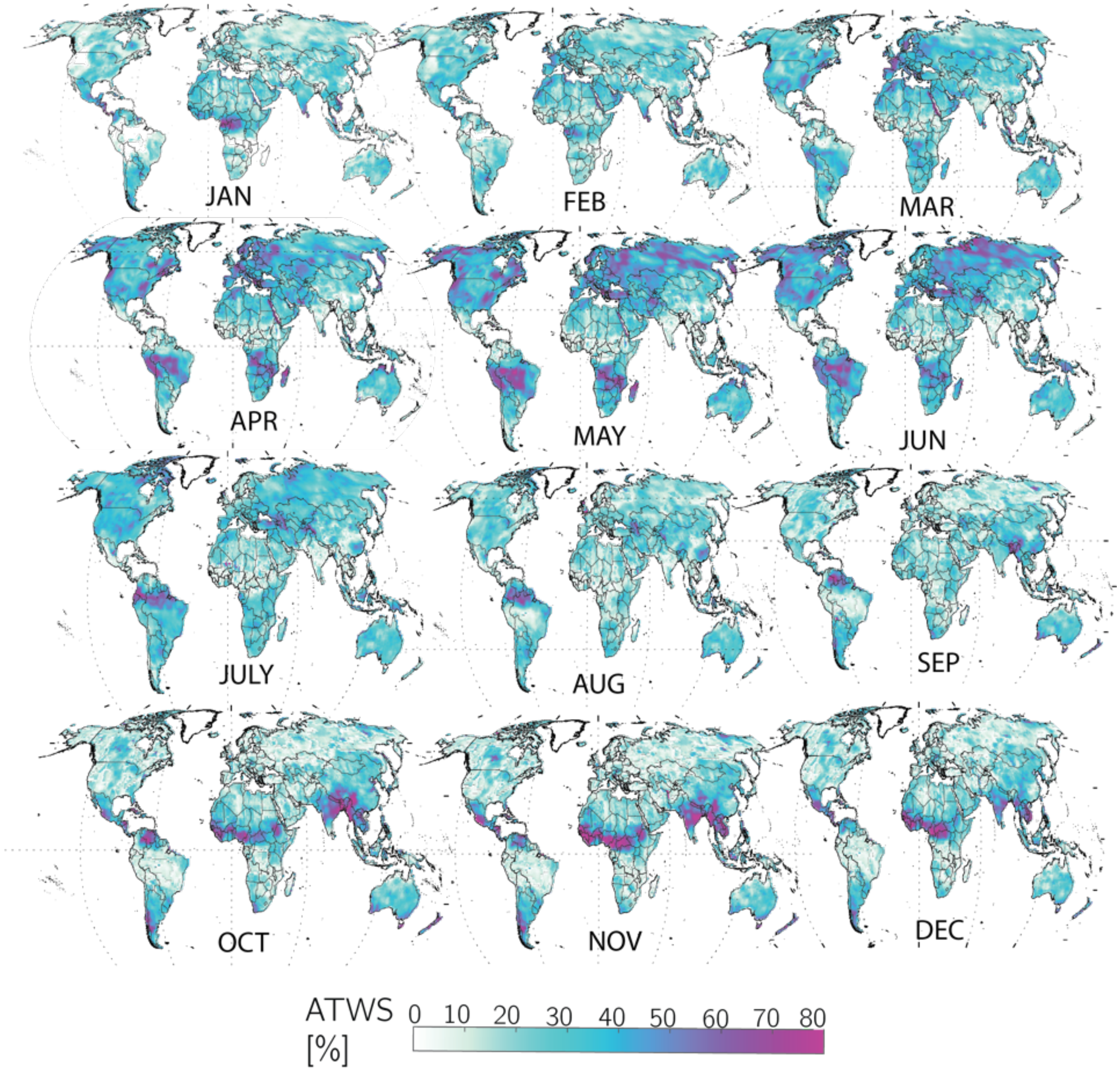

Fig. S13. Climatological mean antecedent total water storage. Data were derived from the 5D solutions mascon solutions between April 17, 2002, and July 1, 2021.

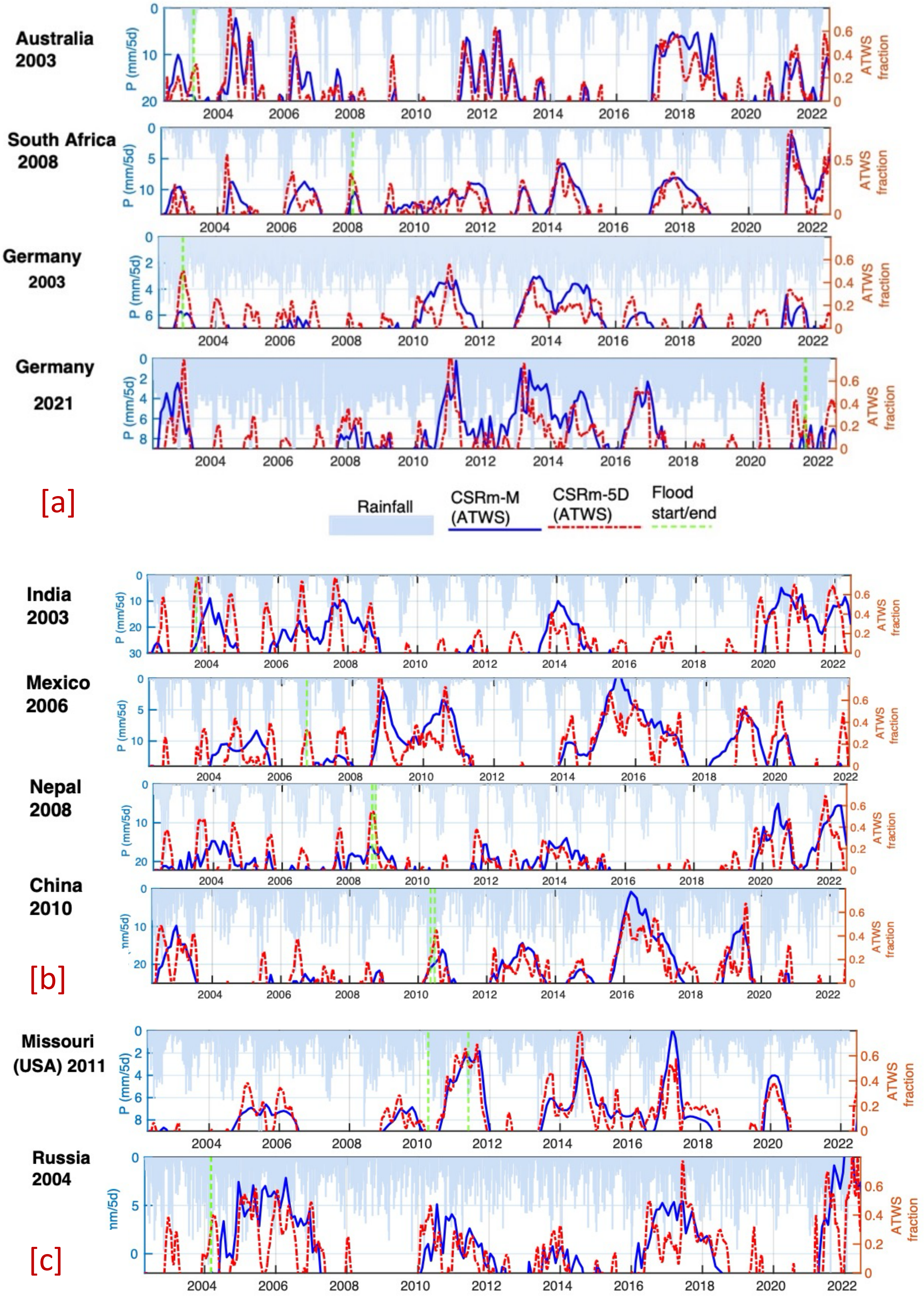

Fig S14. The Antecedent Total Water Storage (ATWS) from the monthly and 5D GRACE and GRACE-FO solutions associated with significant flood events as illustrated in Fig. 1. [a] Heavy rainfall events, [b] monsoon events, and [c] snowmelt events.

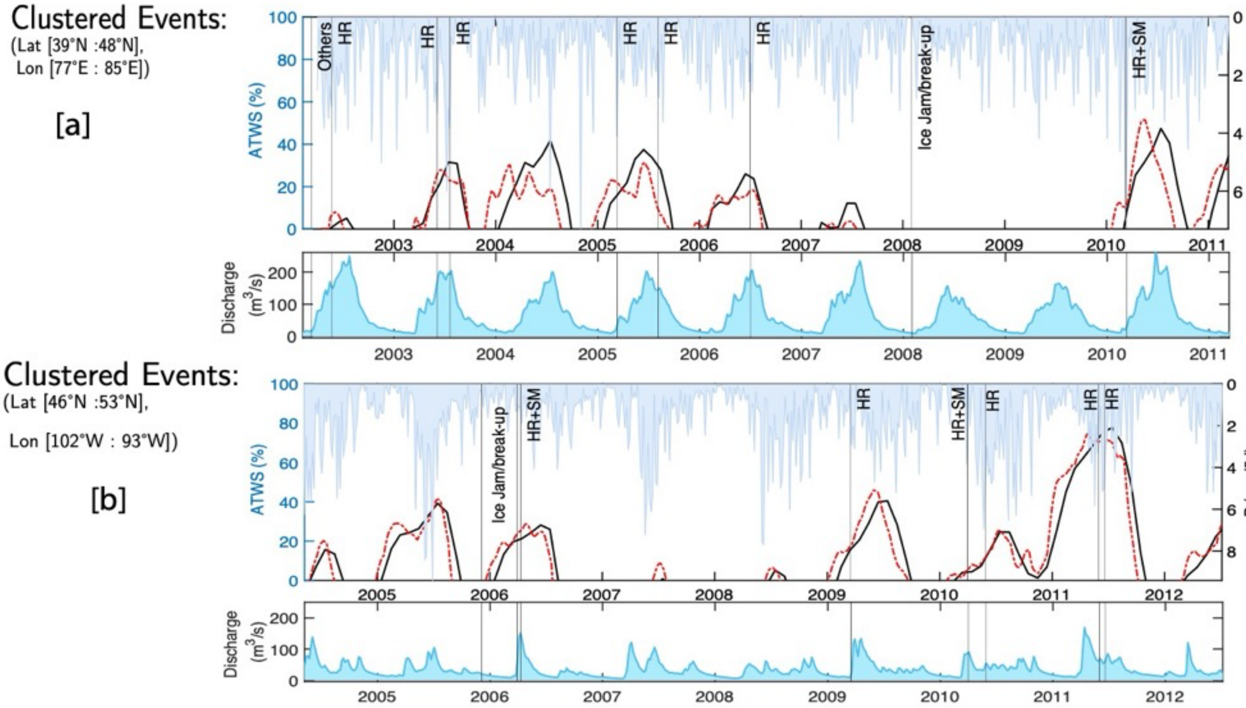

Fig S15: Comparison of precipitation (light blue), Antecedent Total Water Storage (ATWS) from both monthly (black) and 5D (red) solutions, and gridded runoff according to the Global Flood Awareness System (GloFAS). Note the elevated ATWS levels in both the monthly and 5D solutions, with 5D solutions often preceding the monthly ones, which align with higher runoff for various flood mechanisms (SM: snowmelt, HR: heavy rain). However, not every instance of elevated ATWS or increased runoff corresponds with the timing of floods, highlighting the limitations in flood reporting.

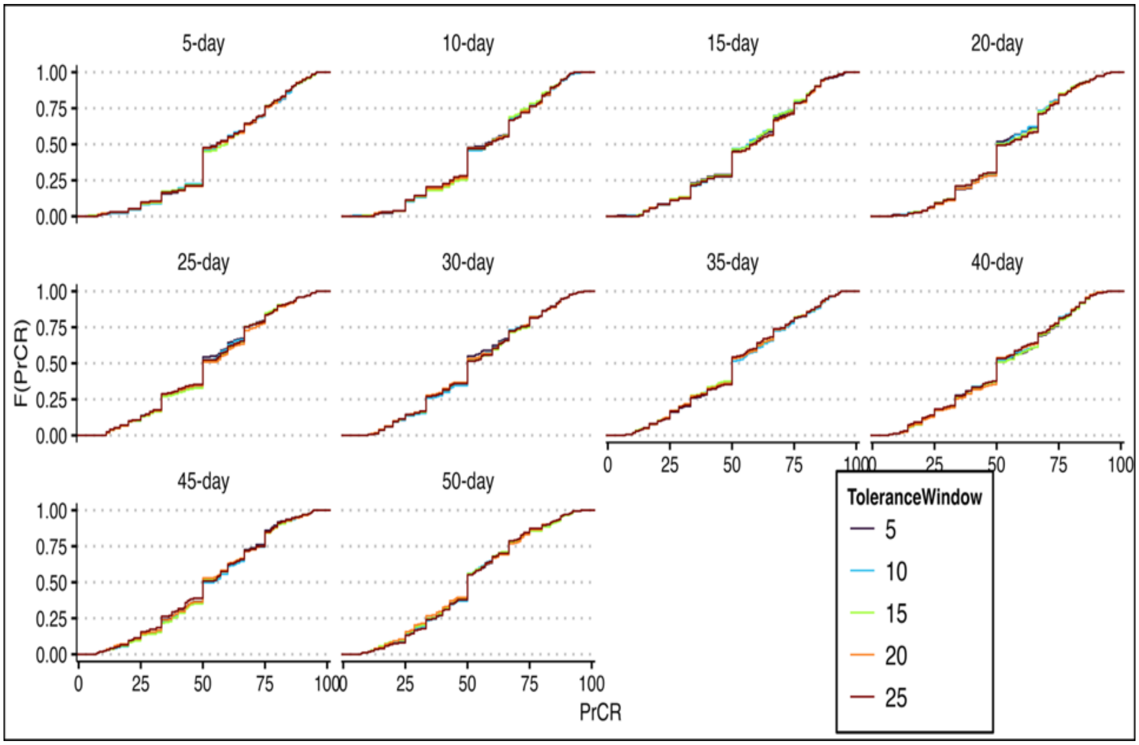

Fig S16. Empirical cumulative density function for the precursor coincidence rates of ATWS preceding 3,272 flood events, considering various lags (i.e., lead times) of ATWS relative to flood duration. The tolerance window illustrates the period during which higher ATWS may occur. Note that the similarity in percentages across all lags is marked, with higher rates observed at shorter lags (5D) and lower at longer lags (50-day). Utilizing a longer tolerance window resulted in an increased precursor coincidence rate (PrCR). More meaningful results were established using a smaller tolerance window (5D).

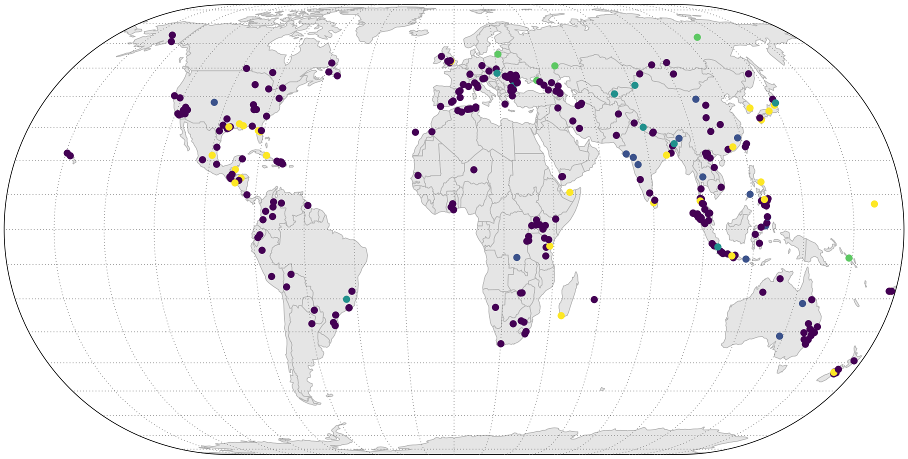

Fig. S17. Locations of flood events not preceded by heightened ATWS. These events are predominantly driven by heavy rain (HR), monsoonal rain (MR), snowmelt (SM), tropical cyclones (TC), and other mechanisms. Such events may be of short duration, occur during droughts, or happen during transitions between dry and wet spells, thereby aiding in storage recovery, or they may be instances where pre-event storage does not play a significant role. Examples of these events are illustrated in Fig.S18.

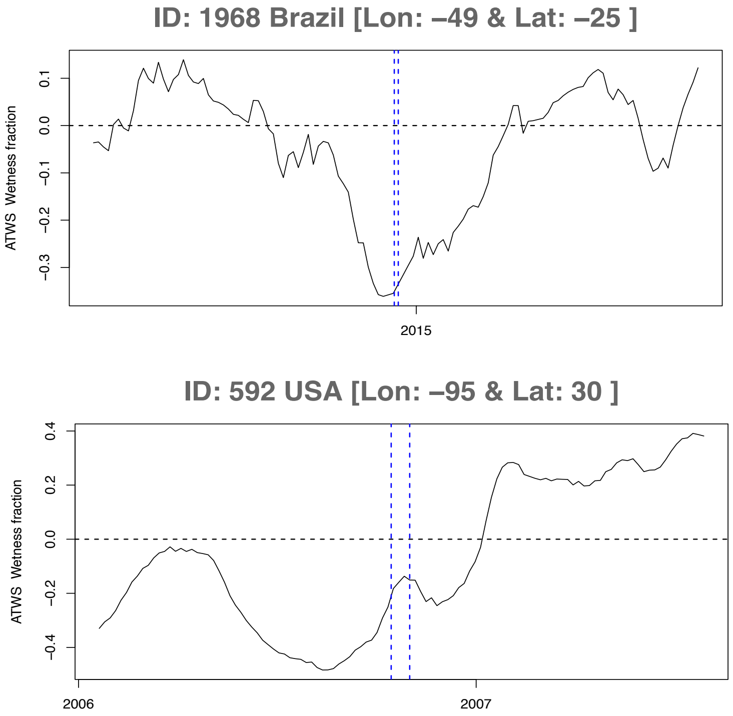

Fig. S18. Two examples of heavy rain flood events that were not preceded by heightened ATWS. One event occurred during a prolonged drought, as observed in Brazil, while the other took place during a transition in storage, aiding in storage recovery, as seen in the USA event.

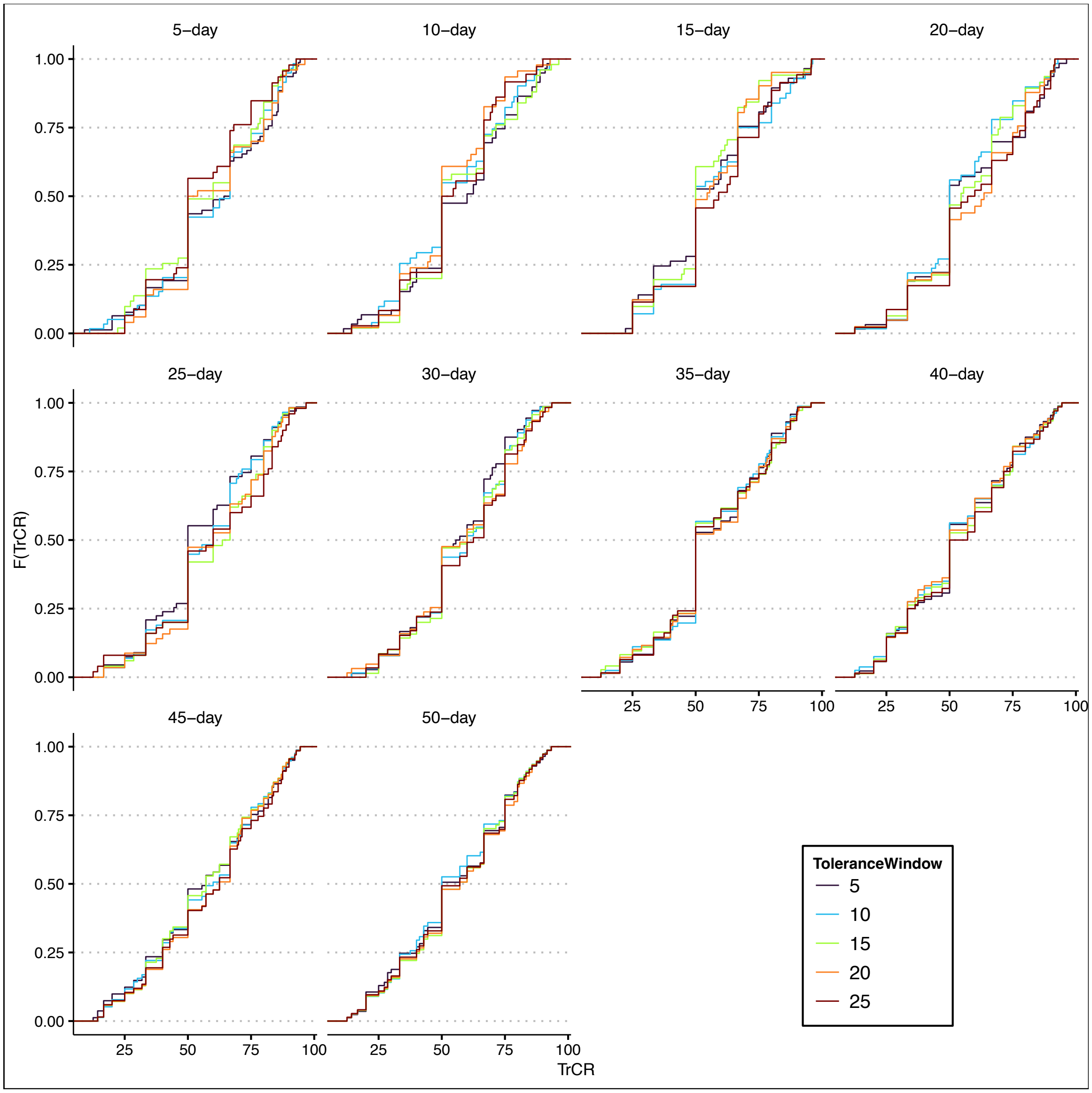

Fig. S19. The empirical cumulative density function for the response coincidence rates of 3,272 flood events that led to above-normal water storage, as detected by the 5D solutions. This analysis accounts for varying lags between the flood events and the increase in ATWS. The tolerance window specifies the number of days within which a flood event can impact storage. "Lag" refers to the time between the onset of an event and its subsequent effect on storage. The most insightful lag, corresponding to rapid storage responses on a submonthly scale (<30 days), is effectively trackable with these data. While varying tolerance windows enhanced the ReCR, they did not affect the lag, indicating that the 5D solution's sampling interval can efficiently monitor significant changes in storage dynamics post-event.

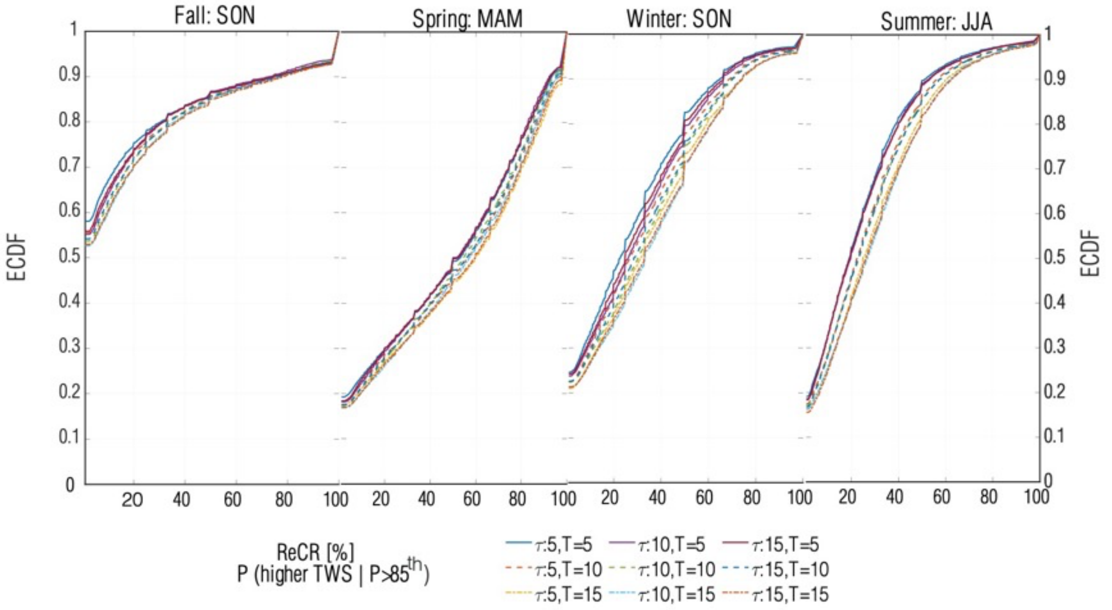

Fig. S20. The empirical cumulative density function of response coincidence rates (ReCR) for intense rainfall events exceeding the 85th percentile, resulting in a positive increase in TWS at the grid scale across all four climate seasons. This association was analyzed considering various lags and tolerance windows. Notably, the proportion of ReCR with extended tolerance windows and the varying rates during each season exhibited dissimilar responses.

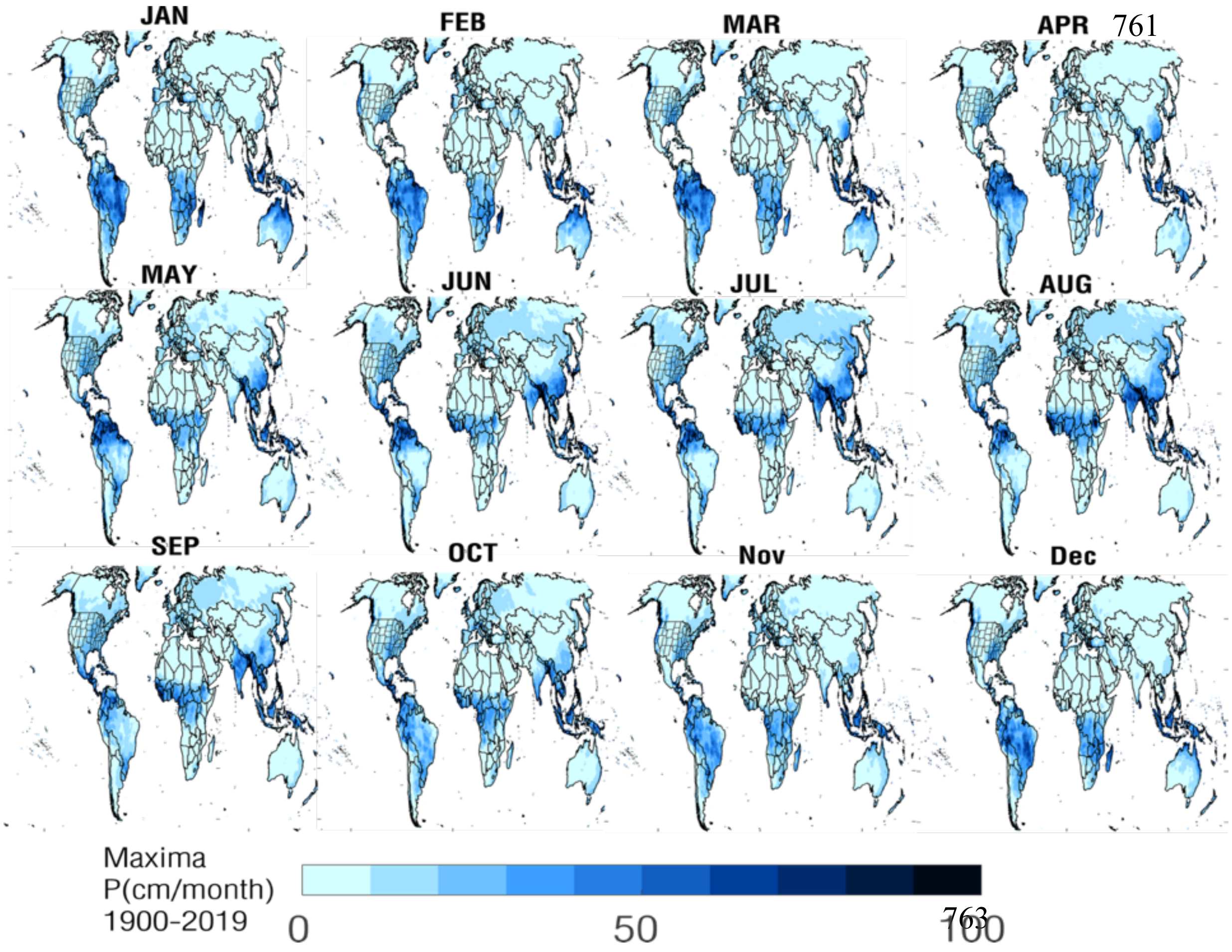

Fig. S21. Monthly maximum precipitation between 1900 and 2019 based on the climate research unit dataset.

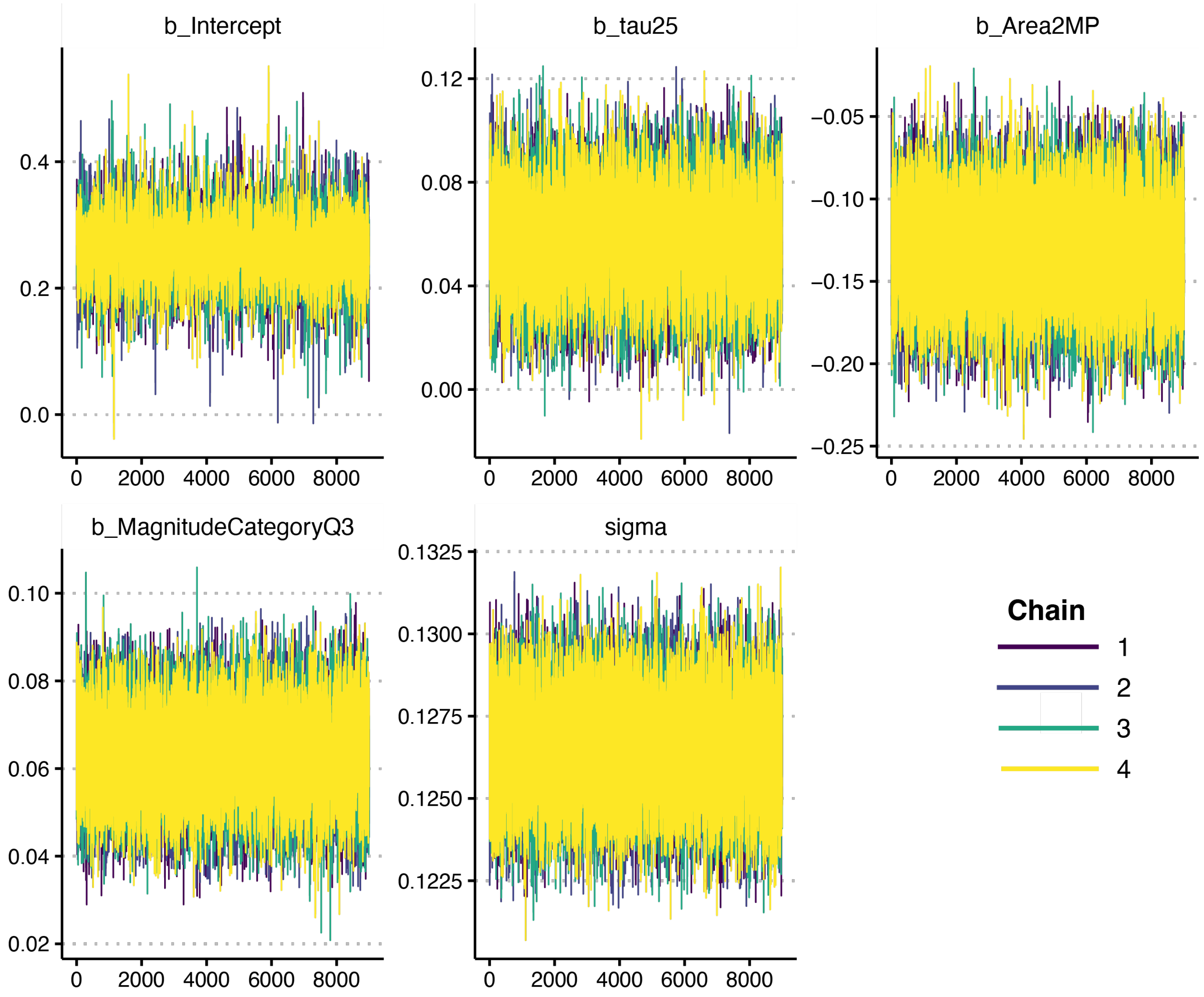

Fig. S22. Markov Chain Monte Carlo (MCMC) trace plots displaying four chains. Each chain includes 15,000 iterations, with the first 6,000 iterations designated as the warm-up phase. The remaining 9,000 iterations are used to generate the posterior distributions of the parameters.

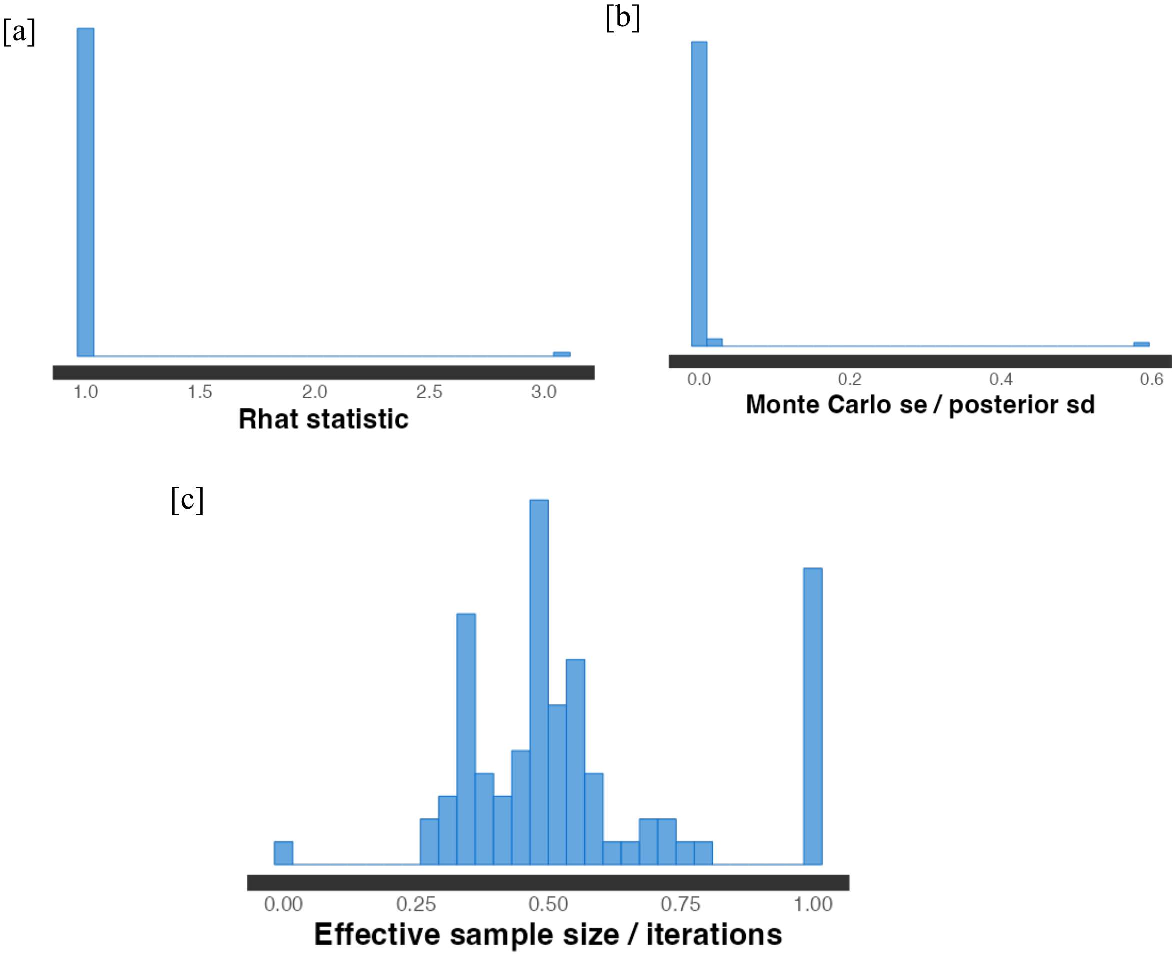

Fig. S23. Diagnostics of Markov Chain Monte Carlo (MCMC) Simulations. [a] $\hat{R}$, commonly referred to as the Gelman-Rubin statistic, is used to assess the convergence of MCMC simulations. It compares the variance within each chain to the variance between chains. A $\hat{R}$ value close to 1 suggests that the chains have converged to the target distribution. [b] Ratio of the Monte Carlo Standard Error (MCSE) to the standard deviation of the posterior distribution. MCSE gauges the uncertainty associated with the Monte Carlo approximation, showing the closeness of the posterior samples to the true posterior distribution. The posterior SD reflects the spread of values within the posterior distribution and measures the uncertainty or variability in the parameter estimates derived from the model. Ratio values near zero suggest that the MCMC simulation efficiently produces precise estimates with minimal error, relative to the inherent variability of the parameter. [c] the effective sample size provides a measure of the number of independent draws from the posterior distribution.

References

1 Kvas, A. *et al.* ITSG-Grace2018: Overview and evaluation of a new GRACE-only gravity field time series. *Journal of Geophysical Research: Solid Earth* **124**, 9332-9344 (2019).

2 Dill, R. *Hydrological model LSDM for operational Earth rotation and gravity field variations*. (GFZ, 2008).

3 Bosilovich, M. G., Lucchesi, R. & Suarez, M. MERRA-2: File specification. (2015).

4 Hersbach, H. *et al.* The ERA5 global reanalysis. *Quarterly Journal of the Royal Meteorological Society* **146**, 1999-2049 (2020).

5 Mallat, S. G. A theory for multiresolution signal decomposition: the wavelet representation. *IEEE transactions on pattern analysis and machine intelligence* **11**, 674-693 (1989).

6 Endres, D. M. & Schindelin, J. E. A new metric for probability distributions. *Ieee T Inform Theory* **49**, 1858-1860 (2003).

7 Fuglede, B. & Topsoe, F. in *International Symposium onInformation Theory, 2004. ISIT 2004. Proceedings.* 31 (IEEE).

8 Eicker, A. *et al.* Daily GRACE satellite data evaluate short-term hydro-meteorological fluxes from global atmospheric reanalyses. *Sci Rep* **10**, 4504 (2020). https://doi.org/10.1038/s41598-020-61166-0

9 Hachborn, E., Berg, A., Levison, J. & Ambadan, J. T. Sensitivity of GRACE-derived estimates of groundwater-level changes in southern Ontario, Canada. *Hydrogeol J* **25**, 2391-2402 (2017). https://doi.org/10.1007/s10040-017-1612-2

10 Webster, P. J. *et al.* Monsoons: Processes, predictability, and the prospects for prediction. *Journal of Geophysical Research: Oceans* **103**, 14451-14510 (1998).

11 Gill, A. E. Some simple solutions for heat-induced tropical circulation. *Quarterly Journal of the Royal Meteorological Society* **106**, 447-462 (1980).

12 Nicholson, S. E. Dryland climatology. (2011).

13 Durbin, J. & Koopman, S. J. *Time series analysis by state space methods*. (Oxford university press, 2012).

14 Gelman, A. *et al. Bayesian data analysis*. (CRC press, 2013).

15 Rateb, A., Sun, A., Scanlon, B. R., Save, H. & Hasan, E. Reconstruction of GRACE mass change time series using a Bayesian framework. *Earth and Space Science* **9**, e2021EA002162 (2022).

16 Williams, C. K. & Rasmussen, C. E. *Gaussian processes for machine learning*. Vol. 2 (MIT press Cambridge, MA, 2006).

17 Carpenter, B. *et al.* Stan: A Probabilistic Programming Language. *J Stat Softw* **76** (2017). https://doi.org/10.18637/jss.v076.i01

18 Bürkner, P. An R package for bayesian multilevel models using Stan. *J Statist Software*
